# Seasonal and ecohydrological regulation of active microbial populations involved in DOC, CO_2_, and CH_4_ fluxes in temperate rainforest soil

**DOI:** 10.1038/s41396-018-0334-3

**Published:** 2018-12-11

**Authors:** David J. Levy-Booth, Ian J. W. Giesbrecht, Colleen T. E. Kellogg, Thierry J. Heger, David V. D’Amore, Patrick J. Keeling, Steven J. Hallam, William W. Mohn

**Affiliations:** 1 Department of Microbiology & Immunology, Life Sciences Institute, University of British Columbia, Vancouver, BC Canada; 2 Hakai Institute, Tula Foundation, Heriot Bay, BC Canada; 3 School of Resource and Environmental Management, Simon Fraser University, Burnaby, BC Canada; 4 The University of Applied Sciences Western Switzerland, CHANGINS, Delémont, Switzerland; 5 U.S. Department of Agriculture, Forest Service, Pacific Northwest Research Station, Juneau, Alaska USA; 6 Department of Botany, University of British Columbia, Vancouver, British Columbia Canada

**Keywords:** Microbial ecology, Biogeochemistry

## Abstract

The Pacific coastal temperate rainforest (PCTR) is a global hot-spot for carbon cycling and export. Yet the influence of microorganisms on carbon cycling processes in PCTR soil is poorly characterized. We developed and tested a conceptual model of seasonal microbial carbon cycling in PCTR soil through integration of geochemistry, micro-meteorology, and eukaryotic and prokaryotic ribosomal amplicon (rRNA) sequencing from 216 soil DNA and RNA libraries. Soil moisture and pH increased during the wet season, with significant correlation to net CO_2_ flux in peat bog and net CH_4_ flux in bog forest soil. Fungal succession in these sites was characterized by the apparent turnover of *Archaeorhizomycetes* phylotypes accounting for 41% of ITS libraries. Anaerobic prokaryotes, including *Syntrophobacteraceae* and *Methanomicrobia* increased in rRNA libraries during the wet season. Putatively active populations of these phylotypes and their biogeochemical marker genes for sulfate and CH_4_ cycling, respectively, were positively correlated following rRNA and metatranscriptomic network analysis. The latter phylotype was positively correlated to CH_4_ fluxes (*r* = 0.46, *p* < 0.0001). Phylotype functional assignments were supported by metatranscriptomic analysis. We propose that active microbial populations respond primarily to changes in hydrology, pH, and nutrient availability. The increased microbial carbon export observed over winter may have ramifications for climate–soil feedbacks in the PCTR.

## Introduction

Soils of the Pacific coastal temperate rainforest (PCTR) of North America sequester globally important amounts of carbon (~198–900 Mg C ha^−1^) [1] and contribute some of the highest rates of dissolved organic carbon (DOC) export to coastal margins in the world (10.5–29.9 g C m^−2^ y^−1^) [2]. Soil CH_4_ fluxes in the PCTR range from uptake (0.05–0.55 mg C m^−2^ h^−1^) in upland forests to strong emissions (0–1.08 mg C m^−2^ h^−1^) from ombrotrophic peat bogs [3]. Microbial communities regulate the flow of carbon through coastal ecosystems via decomposition of plant biomass [4, 5], yet the controls on microbial carbon cycling in hydric soils, such those in the PCTR, are little understood.

Nutrient limitation, low O_2_, and acidic soil in the PCTR restrict organic matter degradation [6]. The carbohydrate-active enzymes (CAZy) database [7] can facilitate investigation of carbon cycling in soil communities [8, 9], and reveal the flow of carbon and energy through peatlands from biopolymer degradation to C1 metabolism [10]. It remains to be seen how environmental conditions in distinct seasons and ecohydrological classes affects microbial organic matter degradation and carbon cycling.

Anaerobic metabolic pathways play a major role in the mineralization of organic carbon in waterlogged soils. Anaerobic degradation in these environments can overcome thermodynamic limitations through the maintenance of low H_2_ concentrations by coupling fermentation by sulfate-reducing bacteria (SRB) to the reduction CO_2_ to CH_4_ by hydrogenotrophic methanogens [11, 12]. This syntrophic interaction is a major component of metabolism in anaerobic bog soil [13], which may be stimulated by winter precipitation in the PCTR. Quantifying carbon balance due to these processes is imperative for reconciling annual terrestrial carbon budgets.

Quantifying active microbial populations can reveal how communities respond to changing environmental conditions and contribute to nutrient cycles. However, limitations of methods of assessing active microbial groups must be addressed. Sufficient mRNA is difficult to extract and purify from high-organic soils. Ribosomal RNA (rRNA) can be recovered using high-throughput methods, but cellular concentration is not well correlated with growth rates in mixed communities [14]. Further, dormant cells can contain detectable rRNA [15]. Yet, rRNA analysis can potentially reduce bias due to dead or dormant cells [16]. As ribosome concentration indicates potential for protein synthesis and thus cellular activity, the analysis of rRNA may provide ecologically-meaningful insights into dynamics of putatively active microbial community members [10, 17–19].

**Fig. 1:**
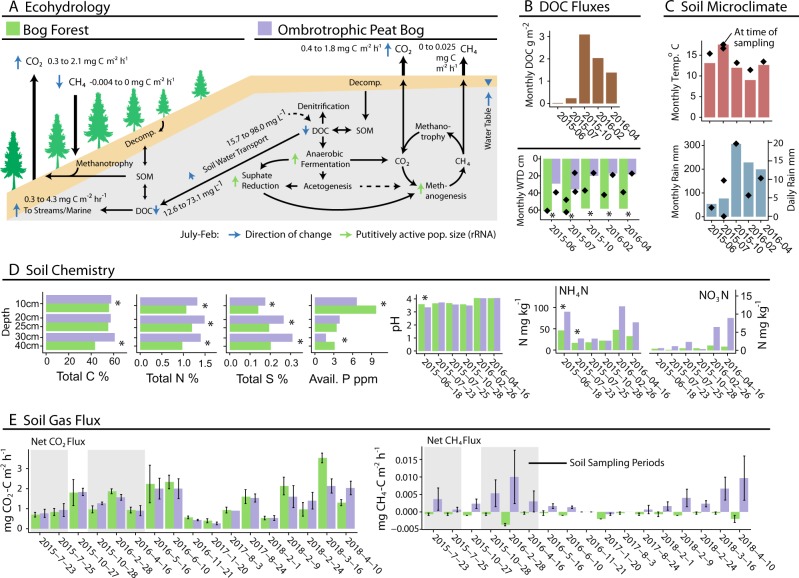
Temperate rainforest ecohydrology and seasonal conditions. **a** Conceptual model of seasonal and ecohydrological effects on carbon flux in a temperate rainforest landscape, including carbon pool and net gas flux ranges. Range (min to max) provided for key measurements. Major changes between dry and wet periods are indicated with arrows shaded by effect type: blue arrow, carbon concentration or flux rate; green arrow, relative abundance of putatively-active microbial groups. Arrows facing up indicate net positive change from dry period to wet period. Ecohydrological sites correspond to typical landscape positions, with bog forests occupying poorly drained slopes and ombrotrophic peatlands occupying flatter sites on higher slope positions. **b** Mean monthly DOC flux from seven adjacent watersheds averaging 6.7 km^2^ (data from [22]). **c** Soil microclimate variables: mean monthly temperature, air temperature at sampling; mean monthly rainfall, 24-h rainfall at sampling, mean monthly water table depth (WTD), WTD at sampling. **d** Soil chemistry variables which did not have a significant seasonal variation component (Supplementary Figure 3) provided by depth and ecohydrology (site). Variables with significant seasonal effects provided as the mean of three depths. **e** Mixed-chamber net soil CO_2_ and CH_4_ flux. Samples corresponding to soil microbial sampling highlighted in gray. Full soil microclimate, chemistry, DOC, and gas flux data, and analysis in Supplementary Figures 2–6. *p* Values following ANOVA are denoted by asterisks for differences between bog forest and peat bog sites at each date or depth (*p < 0.05)

To characterize in situ total (DNA libraries) and putatively active (rRNA libraries) microorganisms and their role in DOC, CO_2_, and CH_4_ cycling and export we sequenced amplicons of archaeal and bacterial 16S rRNA, fungal ITS, and eukaryotic 18S rRNA (focused on soil protists). Metatranscriptomics (mRNA) validated phylotype functional assignment. We hypothesized that soil conditions in distinct ecohydrological classes (peat bog and bog forest) would structure total microbial communities (H1); that putatively active microbial populations would additionally respond to micro-climactic variables in distinct seasons (H2); and that winter periods would increase anaerobic metabolic processes leading to increased net CH_4_ flux (H3). We demonstrated seasonal differences in the structure of putatively active microbial community members, including a response to previously uncharacterized increased pH and inorganic nitrogen concentrations in winter, resulting in enhanced net flux of both CO_2_ and CH_4_. Together, these data allowed us to develop and assess a conceptual model of seasonal changes in microbial carbon cycling in major PCTR ecohydrological classes (Fig. 1a).

## Material and Methods

### Study location and site description

The Calvert Island Field Station is located on an outer-coast island in the Perhumid PCTR (Supplementary Figure 1), in the very wet hypermaritime coastal western hemlock zone (CWH vh2) zone [20]. The bog forest site (TSN2; N51°39′08″, W128°07′47″) is within CWH vh2 site series 11. It contained shallow, nutrient poor organic soils (80–125 cm depth) with distinct L, F, and H layers over Of, Om layers, and some unstructured mineral material. The primary canopy contains *Pinus contorta, Chamaecyparis nootkatensis*, and *Thuja plicata*. The deep, ombrotrophic peat bog site (TSN3; N51°39′05″, W128°07′43″) is in BEC CWH vh2 site series 32. It contained deep (>2 m) peat-derived organic soil, with sparse *P. contorta, C. nootkatensis*, and *T. plicata* and abundant ericaceous shrubs on *Sphagnum* spp. lawns and hummocks. We use the term ecohydrological class for these sites to distinguish areas of discrete vegetation, soil depth, soil type, and hydrology. Peat bog and bog forest sites were chosen on the basis of terrestrial ecosystem mapping methods, to target ecohydrological classes that are prevalent in the PCTR [21].

### Terrestrial sensor nodes

Both sites contained: an HC-S3 air temperature and relative humidity probe and a TB4 rain-gauge controlled with a CR1000 datalogger (Campbell Scientific (Canada) Corp., Edmonton, Canada) and powered by solar arrays. Three subplots per site each contained: three 109-L soil temperature probes (Campbell Scientific (Canada) Corp.), three platinum soil redox potential probes with XR300 Ag/AgCl Reference Electrodes (Radiometer America Inc., Brea, USA). Soil temperature and redox potential probes were placed at depths of 10, 25, and 40 cm in the bog forest site and 10, 20, and 30 cm in the peat bog site, with the depths corresponding to approximate median water table depth and the bounds of annual water table variability.

### Soil, water, and gas sampling and analysis

On June 18, 2015 (early summer), July 23, 2015, July 25, 2015 (summer), October 28, 2015 (fall), February 28, 2016 (winter), and April 18, 2016 (spring), soil was sampled in each subplot at depths corresponding to sensor placement, using a screw auger and peat coring device. Two cores per subplot were pooled to give 216 total soil samples analyzed for this work. Subsamples (5 g) for DNA and RNA extraction were immediately frozen on dry ice. Frozen soil was transferred to a V900 CryoPro Vapor Shipper (VWR International, Radnor, USA) (<−150 °C) for transport and stored at −80 °C. Soil was measured for gravimetric soil water content and pH (1:1 H_2_O), and sent to the British Columbia Analytical Laboratory in Victoria, BC for total C, N, and S, NO_3_-N, NH_4_-N, available P, cation concentrations and effective cation exchange capacity (CEC). Syringe-filtered (20 μm) soil water was sampled at 30 cm depth from pan lysimeters and at 75 cm using piezometers and measured for DOC and specific ultraviolet absorbance at 254 nm. See Supplemental methods and Oliver et al. [22] for details. GC-based measurements of CH_4_ fluxes used large-diameter, 8.4-l fan-mixed polyvinyl chloride chambers as in Christiansen et al. [3]. Totally, 12 ml headspace air was removed every 15 min for 1 h and stored in 6-ml exetainers (LabCo Ltd., Lampeter, Wales). For analysis, 2.5 ml was manually injected into a 5890 Series II gas chromatograph (Agilent Technologies, Santa Clara, USA) equipped with a flame ionization detector and electron capture device.

### Soil nucleic acid extraction and amplicon sequencing

DNA was extracted with PowerSoil DNA Isolation kits (MoBio Laboratories, Inc., Carlsbad, USA) with 0.25 g frozen soil. RNA extraction used a protocol modified from Griffiths et al. [23]: 0.5 g of frozen soil was subject to bead beating twice in lysing matrix E (MP Biomedicals LLC, Santa Ana, USA) containing 0.35 ml 240 mM phosphate buffer (pH 8.0), 0.15 ml 10% CTAB buffer (pH 8.0), and 60 µl of 200 mM AlNH_4_(SO_4_)_2_. Following phenol-chloroform extraction and RNA capture column (MoBio Laboratories, Inc.) purification, DNA was removed with Turbo DNase (Life Technologies Corp., Carlsbad, USA) treatment (confirmed by 16S rRNA qPCR). DNA and RNA concentrations were determined using a Qubit^™^ 3.0 Fluorometer (Thermo Fisher Scientific Inc., Waltham, USA). cDNA synthesis for amplicon library generation used the SuperScript® IV First-Strand Synthesis System (Thermo Fisher Scientific Inc.). Amplification sequencing of prokaryotic 16S-V4 small subunit ribosomal RNA (*SSU* rRNA) and the fungal internal transcribed spacer (ITS) region was conducted by Microbiome Insights Inc. (Vancouver, CAN) using primers 515F/806R [24] following earth microbiome project (EMP) protocols [25]. The fungal ITS V2 region was amplified using primers ITS4/fITS7 with dual-index 8-nt barcodes [26]. Illumina MiSeq V3 300bp-PE sequencing of eukaryotic 18S-V4 SSU rRNA was performed at the Centre for Comparative Genomics and Evolutionary Bioinformatics (Halifax, CAN). A total of 314 DNA libraries and 211 RNA libraries were prepared (Supplementary Table 1). Quality filtering, phylotypes selection and annotation for 16S and 18S rRNA amplicon sequences was informed by EMP guidelines [25] and the open-reference pipeline in QIIME 1.9.1 [27]. Fungal ITS sequence processing used the default PIPITS pipeline [26].

### Metatranscriptomes

Shotgun mRNA sequences were recovered from 10 out of 12, 10 cm samples in July and Oct 2015, including three bog forest and three peat bog samples from July 23, 2015, as well as two bog forest and two peat bog metatranscriptomes from October 28, 2015. Sufficient mRNA was not recovered from deeper samples or from 2016 samples (Supplementary Table 1). Metatranscriptomes were sequenced by The McGill University and Génome Québec Innovation Centre (Montreal, CAN) following application of Ribo-Zero rRNA Removal Kit (Bacteria) (Illumina) using HiSeq2500 125bp-PE. Filtering, clustering and annotation used established pipelines [28].

### Statistical approach

All statistical analysis was performed using *R* 3.2.4. (R Core Team, 2016). Negative binomial normalization of count data using *DeSeq2* 1.18.1 [29] was applied to avoid biases associated with rarefaction [30]. Amplicon sequences were clustered as phylotypes sharing 97% identity. Distance-based redundancy analysis (db-RDA) models were reduced using forward variable selection only if full model *p* < 0.05. Phylotype and KO network correlations were calculated using an ensemble of Compositionality Corrected by REnormalization and Permutation (CCREPE)-corrected Pearson and Spearman correlations (*p* < 0.001) after false discovery rate correction [31] using *ccrepe* 1.12.1. Additional details are provided in Supplementary Methods.

## Results and discussion

### Gaseous carbon fluxes correlated with increasing soil moisture, pH, and nutrient availability during wet periods

Several interconnected seasonal soil micro-meteorological and geochemical trends emerged that were prospectively linked to shifts in microbial community structure and function. High-fall precipitation (306 mm in Oct 2015) (Fig. 1c) elevated the soil water table (Fig. 1c) and gravimetric soil moisture (Supplementary Figure 2). Monthly DOC flux to the marine environment increased from around 0.5–3.35 g m^−2^ between dry and wet seasons in 2015 [22] (Fig. 1b). Soil DOC concentration and aromaticity measurements were inconsistent (Supplementary Figure 3). Toxicity of *Sphagnum*-derived aromatic DOC can suppress microbial activity including sulfate reduction and methanogenesis [32]. Anoxic conditions in bogs due to heavy precipitation can reduce activity of plant-biomass degrading enzymes including phenol oxidases. However, rewetting following drought can counterintuitively stimulate microbial activity in peat soil via depletion of acidic *Sphagnum* DOC along with alleviation of pH stress [6]. Similar DOC flux patterns have been recorded elsewhere in the PCTR [2], with higher and more bioavailable DOC exported from watersheds with a high proportional area of wetlands including bogs [33]. This could indicate differences in quantity and quality of DOC between *Sphagnum* bogs and forests, as well as potential differences in microbial processing.

**Fig. 2:**
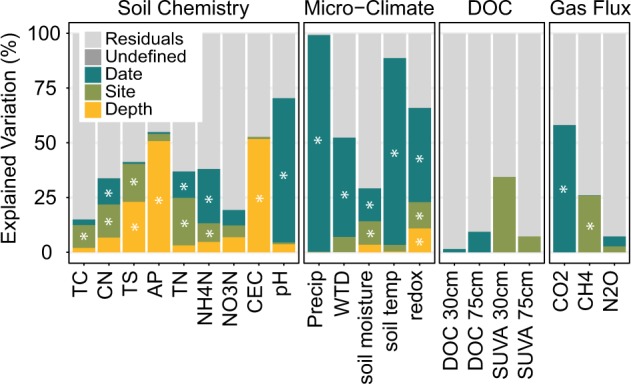
Canonical variation partitioning of soil chemistry, microclimate, soil water DOC, and greenhouse gas flux measurements in the bog forest and peat bog sites by Date, Site and Depth, as well as interaction term (Undefined). FDR-adjusted *p* values following partial regression are denoted by asterisks if significant (*p < 0.05). All data are shown in Supplementary Figures 2–5. TC total carbon (mg/kg), CN C:N ratio, TS total sulfur (mg/kg), AP available phosphate (ppm), TN total nitrogen (mg/kg), CEC cation exchange capacity (CMOL+/kg), WTD water table depth (m), DOC dissolved organic carbon (mg/L), SUVA specific UV absorbance at 254 nm (L/mg/m)

Soil pH rose from 3.35 ± 0.04 in July 23, 2015 to 4.61 ± 0.03 in Feb 28, 2016. Elevated water tables during the wet period (Supplementary Figure 2) likely contributed to the significant temporal component of soil pH variation (68.1%, *p* = 0.001) (Fig. 2), possibly via removal of acidic DOC or depletion of electron acceptors under anaerobic conditions. Soil pH is a major driver of microbial community structure [34, 35]. Alleviation of pH-related stress in acidic forest soils can rapidly increase protein synthesis and growth [36], microbial DOC metabolism [37, 38], and respiration rates in soil bacteria [39]. Increasing pH (e.g., through liming) can reciprocally increase NH_4_-N concentrations in acidic soil [37]. NH_4_-N concentrations also varied between dates (19.4%, *p* = 0.02) (Fig. 1d, Supplementary Figures 4, 5). However, soil redox potential stayed relatively stable (Supplementary Figure 2), responding primarily to depth and precipitation events.

Of the dates with both gas and soil sampling, net CO_2_ flux rates peaked at 2.1 ± 0.17 mg CO_2_-C m^−2^ h^−1^ in Feb 2016 in the bog forest site, an increase of 132.0% over rates on July 23, 2015 (Fig. 1e). Net soil CO_2_ flux rates were significantly different between dates based on linear mixed effects (lme) ANOVA (*p* = 0.0004) (Supplementary Table 2) and canonical variation partitioning (57%, *p* = 0.001) (Fig. 2). In contrast, net CH_4_ flux rate varied by site following canonical variation partitioning (25%, *p* = 0.002) (Fig. 2). When divided by site, net CH_4_ flux rate also displayed a significant date effect in the bog forest site (*p* = 0.0005) (Supplementary Table 2). High net CO_2_ flux rates were also observed during the 2018 wet period (Fig. 1e). Net CH_4_ flux rates in peat bog soil were highest in Feb 2016, with a rate of 0.01 ± 0.011 mg CH_4_-C m^−2^ h^−1^. Bog forests exhibited net atmospheric CH_4_ uptake, which increased 611.6% between July 23, 2015 and Feb 28, 2016. Additional gas flux analysis in 2017 and 2018 shows that CO_2_ and CH_4_ fluxes were highly variable, with higher rates occurring later in the wet season in these years than in 2016. N_2_O fluxes were generally minimal (Supplementary Figure 7). While the contrast between wet and dry season soil respiration appears consistent with a seasonal effect, CO_2_ flux varied substantially within seasons, suggesting a response to rapidly changing weather conditions (i.e., coastal storm events) that have the potential to rapidly alter carbon fluxes in these ecosystems. Importantly, however, the use of 1-h chamber-based flux measurements may miss important sources of variation.

Net gas fluxes appeared to respond to different factors across ecohydrological sites. Bog forest CO_2_ flux variation was explained by pH (28.8%, *p* = 0.001) and redox potential (3.4%, *p* = 0.038), while peat bog CO_2_ flux variation was explained by soil moisture (65.0%, *p* = 0.001), total carbon (16.5%, *p* = 0.001), available phosphorus (5.2%, *p* = 0.001), and NO_3_-N (4.2%, *p* = 0.007) (Supplementary Figure 6A). In nonhydric temperate soils, high-soil water content generally suppresses respiration [40]. Bog forest CH_4_ flux variation was explained by pH (36.7%, *p* = 0.001) and NO_3_-N (2.5%, *p* = 0.047), and peat bog CH_4_ flux variation by soil moisture (45.8%, *p* = 0.001), water table depth (12.3%, *p* = 0.002) (Supplementary Figure 6B). These data suggest functional changes in the bog forest site due to pH, and in the peat bog site due to hydrology, with nutrient availability having minor effects.

In temperate bog and fen soils, respiration and CH_4_ fluxes increased when pH was increased in the range observed in this study (~3.5–4.5) with CH_4_ fluxes being 436% more sensitive to pH increase than CO_2_ fluxes [41]. Although, others found little effect of pH on soil respiration [42]. While we did not directly measure the contribution of plants to microbial activity or soil gas flux, belowground carbon allocation by plants contributes to respiration rates [43] and seasonal CH_4_ flux rates [44]. Further, seasonal changes in temperate forest resource allocation can shift soil microbial activity in winter toward degradation of complex carbon substrates [45]. While PCTR CH_4_ fluxes are poorly characterized, seasonal flux rates can be weakly correlated to the ratio of total methanotoph *pmoA* gene copies to methanogen *mcrA* gene copies across an ecohydrological gradient (*R*^2^ = 0.21) [3], and, strongly to water table depth (*R*^2^ = 0.78) [46].

Nitrogen can have variable effects on net methane flux rates in soil. Inorganic forms can inhibit or stimulate both methanotrophy [47] and methanogenesis [48–51]. NH_4_-N can inhibit methanotrophy [52] by being selectively bound by the PMO enzyme in place of CH_4_ [53]. NO_3_-N can inhibit acetoclastic methanogenesis due to competition from denitrifers [54]. However, nitrogen availability in soils can stimulate methane production or oxidation due to the alleviation of limitations, e.g., on protein synthesis, or switching from “metabolically expensive” nitrogen fixation to endogenous nitrogen resources [47, 49, 55, 56]. In nitrogen-limited peatlands, experimental nitrogen addition can increase net CO_2_ and CH_4_ flux rates [57]. In our soil system NO_3_-N was weakly correlated to net CH_4_ flux rates. The seasonal dynamics of methane cycling populations can potentially reveal the mechanism of nitrogen effects on net CH_4_ fluxes.

### Microbial communities in rRNA-libraries shift with microclimate changes and nutrient availability in contrasting seasons

**Fig. 3:**
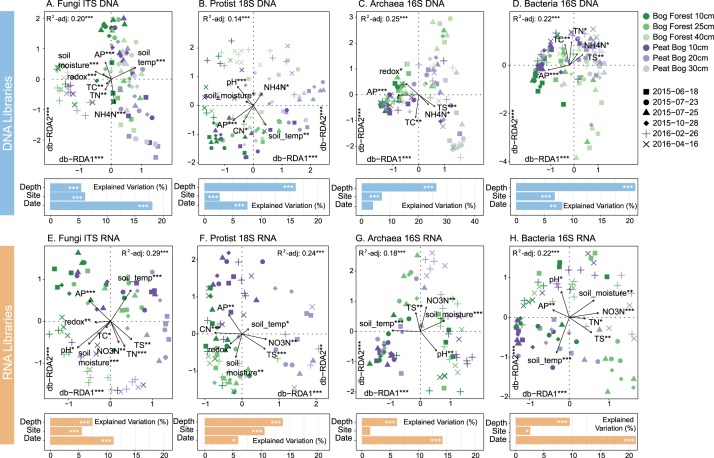
Distance-based redundancy analysis (db-RDA) of Bray–Curtis dissimilarly for **a** fungal ITS, **b** protist 18S rRNA gene, **c** archaeal 16S rRNA gene and **d** bacterial 16S rRNA gene phylotypes in DNA libraries, and **e** fungal ITS, **f** protist 18S rRNA, **g** archaeal 16S rRNA, **h** bacterial 16S rRNA in RNA libraries at Bog Forest (TSN2) and Peat Bog (TSN3) sites constrained by soil chemistry and climate variables. Model and axis significance determined by RDA ANOVA. Variable significance (bargraphs) was determined by PERMANOVA, shown as adjusted-*R*^2^ following PERMANOVA by categorical variables. *p* Values are denoted by asterisks (*p < 0.05, **p < 0.01, ***p < 0.001). TC total carbon, TN total nitrogen, TS total sulfur, CN carbon:nitrogen ratio, AP available phosphorus

Microbial communities extrapolated from DNA and RNA amplicon libraries differed following PERMANOVA, with the highest dissimilarity observed for bacterial 16S rRNA libraries (*R*^2^ = 0.15, *p* = 0.001) and lowest for fungal ITS libraries (*R*^2^ = 0.05, *p* = 0.001) (Supplementary Figures 8 and 9). Extraction protocols did not appear to influence community composition (Supplementary Figure 10). Total C, available P, and NH_4_-N were significant sources of variation following regression of total fungal, protist, archaeal and bacterial Bray–Curtis dissimilarity matrices (Fig. 3). Eukaryotic and prokaryotic community structure in DNA libraries were also influenced by micro-climactic conditions and total S, respectively. Depth was a significant source of variation in all communities and library types, with site as a secondary factor. These results support the expectation that ecohydrological sites would support distinct microbial communities (H1). All communities in RNA libraries were significantly structured by date. This observation supports H2, as potentially active community structure was influenced by temporal variations to a greater extent than total community structure from DNA libraries.

**Fig. 4:**
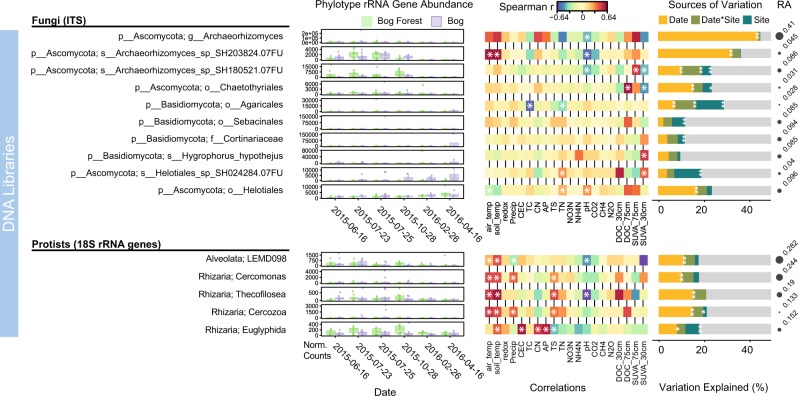
Influence of season and ecohydrology on fungal ITS and Protist 18S rRNA phylotype abundance in DNA libraries. DeSeq2-normalized counts for the ten most-abundant fungal phylotypes and five most-abundant Protist phylotypes in bog forest and peat bog sites shown for each sampling date. Spearman correlations with environmental variables and variation partitioning using partial regression results provided for each phylotype. FDR-corrected *q* values < 0.1 following Spearman correlation are denoted by a single asterisks. Sources of variation following partial regression results are denoted by asterisks according to *p* value (*p < 0.05, **p < 0.01, ***p < 0.001). Circle size shows phylotype relative abundance (RA) in DNA libraries. Fungal ITS and protist 18S rRNA phylotype abundance in RNA libraries shown in Supplementary Figure 10

To further understand seasonal dynamics of highly abundant microbial taxa, the 5% most-abundant fungal, protist, archaeal, and bacterial phylotypes (66% of sequence counts) were tested for variation sources and environmental correlations. Fungal ITS communities consisted of few, highly abundant taxa. *Archaeorhizomycetes* accounted for 41% of all ITS counts following *DeSeq2* normalization, yet decreased 99.4% in DNA libraries from Oct to April (Fig. 4). Season explained 44.1% (*p* = 0.002) and 36.2% (*p* *=* 0.002) of variation in *Archaeorhizomycetes* and *Archaeorhizomycetes* SH203824.07FU phylotype abundance, respectively. Abundance of SH203824.07FU was positively correlated to soil temperature (*r* = 0.54, *p* < 0.0001) and negatively to pH (*r* = −0.31, *p* = 0.002), indicating adaptation to habitats with pH < 4.0. Similar trends were observed in rRNA libraries (Supplementary Figure 11). *Archaeorhizomycetes* are probable saprotrophs found in conifer rhizospheres and are able to metabolize simple sugars, plant root exudates, and cellulose [58]. Their previously described seasonality [59] could be linked to alterations in soil DOC content as well as plant growth, exudation, and biomass degradation. In contrast, putatively saprotrophic *Ascomycetes* in the order *Helotiales*, which have been shown to be abundant in arable soils with pH < 5.0 [35], appeared to respond positively to pH, indicating possible pH-driven seasonal succession in fungal communities. Nonfungal Eukaryotes are an oft-neglected component of the total microbial community. The high proportion of bacterial-feeding rhizarians was described previously in the *Sphagnum* moss cover of coastal bogs [60]. The potential seasonal reduction in protist groups could have repercussions throughout the microbial food web due alleviation of predation over winter.

**Fig. 5:**
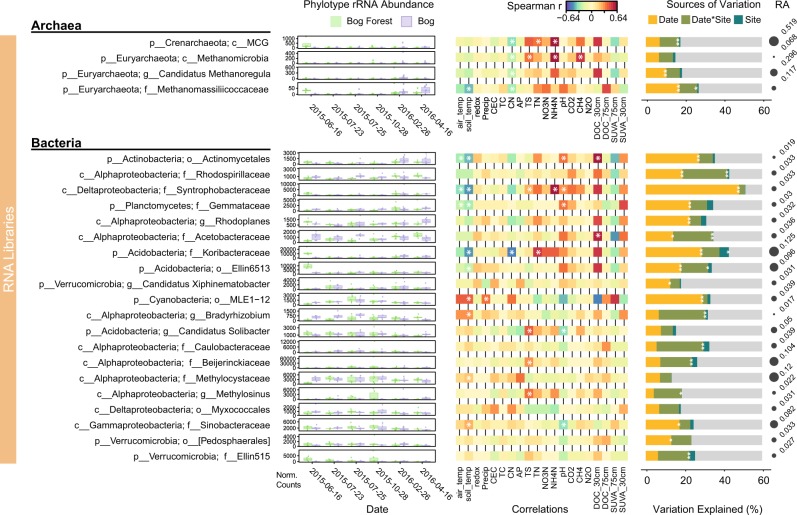
Influence of season and ecohydrology on archaeal and bacterial 16S rRNA phylotype abundance in RNA libraries. DeSeq2-normalized counts for the 4 most-abundant archaeal phylotypes and 20 most-abundant bacterial phylotypes in bog forest and peat bog sites shown for each sampling date. Spearman correlations, variation partitioning and as in Fig. 4. Circle size shows phylotype relative abundance (RA) in DNA libraries. Archaeal and bacterial rRNA gene phylotype abundance in DNA libraries shown in Supplementary Figure 11

Abundance of 14 of the top 24 prokaryotic phylotypes in RNA libraries increased during wet periods in response to pH, nitrogen concentrations, and DOC concentrations (Fig. 5). Phylotypes showing seasonally dynamic putative activity include groups carrying out the linked reduction of SO_4_ and production of CH_4_: *Syntrophobacteraceae*, *Methanoregula*, and *Methanomassiliicoccaceae* [13, 61–64]. Abundance of active *Methanomicrobia* was positively correlated to NH_4_-N concentration (*r* = 0.50, *p* < 0.0001), and to CH_4_ efflux rates (*r* = 0.46, *p* < 0.0001). The proportional increase in sulfate-reducing and methanogenic taxa with inorganic nitrogen could indicate direct or indirect stimulation of these groups. The mechanism elevating NH_4_-N concentrations during the wet season are unknown. Waterlogged peat soils are thought to suppress nitrogen mineralization [65] and nitrification [66]. It remains to be seen if the proposed die-off of *Archaeorhizomycetes* and other Eukaryotic microorganisms provides a source of NH_4_-N during winter.

Archaeal and bacterial phylotypes in DNA libraries did not display significant seasonal effects, with the abundance of only one of the top 24 phylotypes significantly influenced by season (Supplementary Figure 12). Bacterial phylotypes in DNA libraries show near-unanimous positive correlations with redox potential, CEC, CN ratio, and available PO_4_-P, supporting db-RDA results (Fig. 3). This demonstrates that seasonal changes in PCTR soils do not greatly affect the total prokayrotic community composition. In support of H3, which predicted increased anaerobic metabolism during wet periods, it is likely that changes in soil function could be associated with changes in the proportion of putatively active community members including anaerobic bacteria and archaea, rather than total community compositional shifts.

**Fig. 6:**
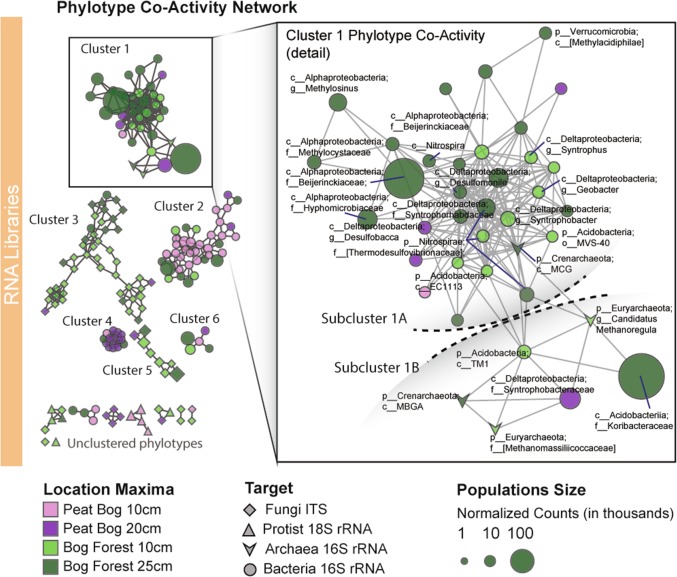
Phylotype co-activity network of fungal ITS, protist 18S rRNA, archaeal 16S rRNA and bacterial 16S rRNA phylotypes in RNA libraries. **a** Node shape indicates taxonomic group and color indicates phylotype location preference (ecohydrology and soil depth of maximum OTU abundance). Node size scaled by DeSeq2-normalized abundance counts. Only taxa were found in DNA and RNA libraries in all habitats and dates were included in the network. Bog Forest co-activity cluster 1 shown in detail in inset. Phylotype environmental correlations are shown in Supplementary Figure 13

Network analysis of microbial communities can elucidate potential metabolic interactions [67, 68] or habitat preferences [69]. Positive correlation of putatively active microbial populations, “co-activity”, does not necessitate biological interaction, although it can determine what biological interactions are possible [70]. Phylotypes show distinct clustering by the depth and ecohydrological site in which each taxon was maximally abundant (Fig. 6). Clusters 2 and 6 contained diverse heterotrophic *Alphaproteobacteria*, *Acidobacteria*, *Bacteroidetes*, *Verrucomicrobia*, and several *Burkholderia* species. Clusters 3 and 5 were comprised of loosely correlated fungal phylotypes including *Archaeorhizomyces*, with cluster 5 differentiated by positive correlation with available phosporus (Supplementary Figure 13). Cluster 4 included strongly correlated *Enterobacteriaceae* and *Clostridia*, positively correlated with pH. Cluster 1 could be divided into two subclusters that respectively contained the two largest bacterial populations: *Beijerinckiaceae* and *Koribacteraceae* (10.4% and 12.5% of bacterial rRNA reads, respectively).

The *Beijerinckiaceae*, which includes obligate and facultative methanotrophs [71, 72], are positively correlated with other closely related alphaproteobacterial obligate aerobic methanotrophs: the *Methylocystaceae* (*r* = 0.84, *p* < 0.0001), including *Methylosinus* sp. (*r* = 0.72, *p* < 0.0001) [73]. Some members of families *Beijerinckiaceae* and *Methylocystaceae* are able to grow on short-chain fatty acids (SCFA) [72, 74] in addition to CH_4_. Both gammaproteobacterial and alphaproteobacterial methanotrophs are abundant and active methane oxidizers in peat soils [75]. Both groups were detected in PCTR soil, but alphaproteobacterial methanotrophs were 6.3 × 10^3^ and 1.2 × 10^5^ times more abundant than gammaproteobacterial methanotrophs (e.g., *Methylomonas* sp.) in DNA and RNA libraries, respectively. Dominant methanotrophs had relatively static populations, with relative abundances in RNA libraries exceeding those in DNA libraries (Supplemental Figure 11). *Beijerinckiaceae* also correlated with *Hyphomicrobiaceae* (*r* = 0.75, *p* < 0.0001), which exhibit slow, aerobic growth on acetate, pyruvate or C1 compounds [76], as well as several groups of SRB including *Syntrophobacteraceae* [77, 78]. These organisms reside in subcluster 1A, apparently capable of a mix of dissimilatory sulfate-reducing or aerobic C1/SCFA metabolism, highlighting an apparent major biological pathway of carbon and energy flow through PCTR soil ecosystems.

*Koribacteraceae* positively correlated with *Syntrophobacteraceae* (*r* = 0.64, *p* < 0.0001), with the hydrogenotrophic/CO_2_ methanogenic genus *Methanoregula* (*r* = 0.67, *p* < 0.0001) [61], and with *Methanomassiliicoccaceae* (*r* = 0.61, *p* < 0.0001), including members that can reduce methanol to CH_4_ [64, 79]. The metabolism of *Koribacteraceae* is not well-understood [80], but sequenced genomes reveal acidophilic organisms containing cellulases, hemicellulases, polysaccharide lyases, and pectin esterases suitable for plant-biomass degradation. While *Koribacter* sp. carbon monoxide oxidation is a hypothesized energy source under thermodynamically limited conditions [81], no *Koribacter* carbon monoxide dehydrogenase transcripts were detected in soil metatranscriptomes. Due to their population size and position within the network, it is possible that *Koribacteraceae* play an undefined role in coupling biopolymer degradation to hydrogenotrophic methanogenesis. The positive correlation between *Syntrophobacteraceae* and hydrogenotrophic *Methanomicrobia* (*r* = 0.92, *p* < 0.0001) likely reflects syntrophic metabolism [13]. The dominant phylotypes in PCTR soil are abundant and widespread in peat soil [10, 82–85], highlighting that the potential ecological relationships important to organic carbon metabolism at our sites are likely shared with acidic peat ecosystems throughout the world.

Metatranscriptomic analysis was used to validate taxon-based functional assignment of phylotypes and elucidate potential functional consequences of populations shifts found in the DNA and RNA amplicon libraries. The high DOC content of PCTR soil (up to 98 mg L^−1^) prevented mRNA extraction and purification from many samples (See Supplementary Table 1 for mRNA sample origins). About 2.6% of the 23.8 million unique transcripts were annotated as CAZy families (Supplementary Figure 13), while CAZymes accounted for about 0.6–0.8% of reads in similar bog systems [86], highlighting the importance of organic decomposition in these soil communities. Forest soil metagenomes from throughout North America had an average CAZy family richness of 235 in metagenomes [9], while Russian peat metatranscriptomes had a richness of 226 of the equivalent CAZy families [10]. Our soil metatranscriptomes had a CAZy richness of 259 families. Fungal GMC oxidoreductases (AA3) were the most abundant of the AA class transcripts (31–35%), lower than their 51–65% relative abundance upland forest metagenomes [9]. Bacterial expression of AA1 laccase, ferroxidase and multicopper-oxidase was observed, suggesting that bacteria have substantial capacity for oxidative decomposition in these soils. 1,4-benzoquinone reductase (AA6) and Cu-dependent lytic polysaccharide monooxygenases (LPMOs) (AA10) enzymes showed high sequence similarity to those in *Ascomycota*, *Plantomycetes*, and *Acidobacteria* (including *Koribacteria* sp.). AA6 and AA10 are capable of oxidizing aromatic compounds [87], and chitin and cellulose [88], respectively. Highly expressed AA11 LPMOs were primarily assigned to filamentous *Ascomycota* including *Neurospora crassa* and *Thielavia terrestris*, which are capable of hydrolyzing all major polysaccharides found in biomass [89, 90]. Actinobacterial transcripts were abundant in CAZy profiles (Supplementary Figure 13) despite the low relative abundance of Actinobacteria in rRNA profiles (Supplementary Figure 10). *Archaeorhizomycetes* are poorly represented in nonredundant protein libraries [91]. Nevertheless, these data support the previous finding that acidic peat bogs and coastal forest litter selects for predominantly *Ascomycota*, *Actinobacteria, Acidobacteria*, and *Alphaproteobacteria* biomass degraders [10, 17, 19, 86].

**Fig. 7:**
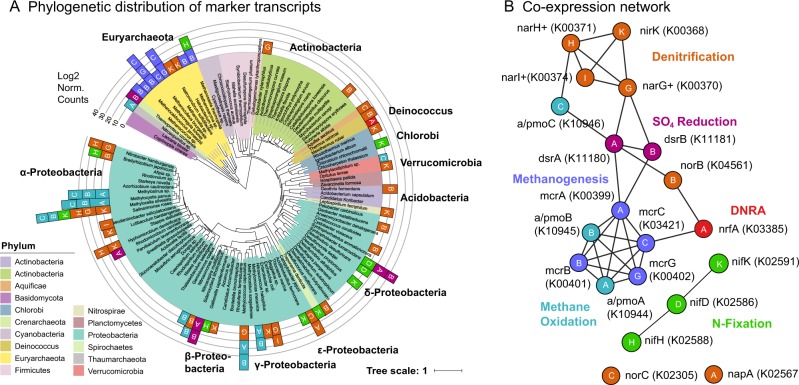
CH_4_, N, and S metabolism gene expression in peat bog and bog forest soil metatranscriptomes (depth: 10 cm). **a** Phylogenetic distribution of selected markers for CH_4_, N, and S metabolism with DeSeq2-normalized transcript abundance counts mapped to 16S rRNA phylogenetic trees. **b** Co-expression network showing correlations between marker transcripts (*p* < 0.001). Genes followed by a + have multiple names for a particular KO. KO IDs for phylogenetic tree provided in network panel. DNRA dissimilatory nitrate reduction to ammonia. Full, interactive CH_4_, N, and S metabolism marker tree (https://itol.embl.de/tree/20687130140153041514909028)

Transcripts that serve as “markers” for key soil geochemical transformations are denoted by their KEGG ortholog (KO) number (Fig. 7a). Only 7.4% the 6.1 million reads mapping to 11 202 KOs could be annotated to species level. Of these, transcripts in the nitrogen-fixation operon (*nifDHK*) were most abundant from the *Beijerinckiaceae* methanotroph *Methylocella silvestris* (*n* = 14), the metabolically diverse methanogen *Methanosarcina* (*n* = 14) and the SRB *Syntrophobacter fumaroxidans* (*n* = 12), the latter of which was also associated with sulfite reductase (*dsrAB*) expression (*n* = 19). Particulate methane monooxygenase (*pmoABC*) transcript sequences most closely aligned with alphaproteobacterial *Methylocystis parvus* (*n* = 14) and *Methylosinus* sp. (*n* = 13), and with gammaproteobacterial *Methylococcus capsulatus* (*n* = 36) and *Methylomonas methanica* (*n* = 2). Methyl-coenzyme M reductase (*mcrABGC*) operons were expressed in *Methanosarcina* (*n* = 4) and in the hydrogenotrophic *Methanocella conradii* (*n* = 29) and *Methanoregula boonei* (*n* = 14). Denitrification (*n* = 199) genes were phylogenetically diverse. Abundant *Gammaproteobacteria* methanotroph transcripts were identified, in contrast with their low detection in ribosomal amplicon libraries. Metatranscriptomic analysis otherwise confirmed assumptions of the metabolic functions of key phylotypes. Similarly, correlations between taxa (Fig. 6) are reflected in the transcript co-expression network (Fig. 7b), showing strong positive correlations between *pmoABC*, *mcrABGC*, and *dsrAB*, and with denitrification genes. The abundance of transcripts in CO_2_-, N-, and S-reduction pathways at 10 cm depth reflects substantial cryptic methanogenesis [92] and other anaerobic processes [93] that can occur in oxic soil.

In this study, we demonstrated a seasonal response of PCTR peat bog and bog forest soil communities. Seasonal turnover of fungi and protists co-occurs with decreasing temperature as well as increasing precipitation, pH and nutrient availability. These biotic and abiotic shifts appear to stimulate anaerobic activity, e.g., by SRB and methanogenic phylotypes, associated with changes in net CH_4_ flux rates. While this study does not account for the potentially important effects of rapid storm-driven changes in microbial activity and carbon flux, it shows that rRNA amplicon sequencing, alongside rRNA gene amplicon and mRNA characterization, can link seasonal shifts in soil conditions and ecosystem function to the abundance of putatively active microbial phylotypes. Further, changes in climactic patterns that alter these biotic and abiotic interactions could alter the fate of carbon including DOC, CO_2_, and CH_4_ fluxes from coastal temperate rainforest soils.

## Supplementary information

Supplemental Figures

Supplemental Methods

## Data Availability

All metadata are archived in the Hakai Institute data repository at 10.21966/1.715630. All raw amplicon reads can be found in the European Nucleotide Archive (ENA) (http://www.ebi.ac.uk/ena) with accessions: 16S rRNA (ERS1798478–ERS1798770), ITS (ERS1798771–ERS1799064), and 18 S rRNA (ERS1799065–ERS1799358). Raw metatranscriptome reads are in ENA accessions ERS1799437–ERS1799448.
