# Supplementary material for: Seasonal and ecohydrological regulation of active microbial populations involved in DOC, CO2, and CH4 fluxes in temperate rainforest soil
Source: ISME J. 2018 Dec 11;13(4):950–63. doi: 10.1038/s41396-018-0334-3 (PMC6461783; doi:10.1038/s41396-018-0334-3)
Supplement: Supplementary file 1 — Supplemental Figures [file 41396_2018_334_MOESM1_ESM.pdf]

Supplementary Material: Seasonal and ecohydrological regulation of active microbial populations involved in DOC, CO_2 and CH_4 fluxes in temperate rainforest soil

David J. Levy-Booth[1,2], Ian J.W. Giesbrecht[2,3], Colleen T.E. Kellogg[1,2], Thierry J. Heger[4], David V. D'Amore[5], Patrick J. Keeling[6], Steven J. Hallam[1], William W. Mohn[1]*

Affiliations:

1. Department of Microbiology & Immunology, Life Sciences Institute, University of British Columbia, Vancouver, British Columbia, Canada
2. Hakai Institute, Tula Foundation, Heriot Bay, British Columbia, Canada
3. School of Resource and Environmental Management, Simon Fraser University, Burnaby, British Columbia, Canada
4. The University of Applied Sciences Western Switzerland, CHANGINS, Switzerland
5. U.S. Department of Agriculture, Forest Service, Pacific Northwest Research Station, Juneau, Alaska, USA
6. Department of Botany, University of British Columbia, Vancouver, British Columbia, Canada

*Correspondence: WW Mohn, Department of Microbiology & Immunology, Life Sciences Institute, University of British Columbia, 2350 Health Sciences Mall, Vancouver, British Columbia V6T 1Z3, Canada.

E-mail: wmohn@mail.ubc.ca

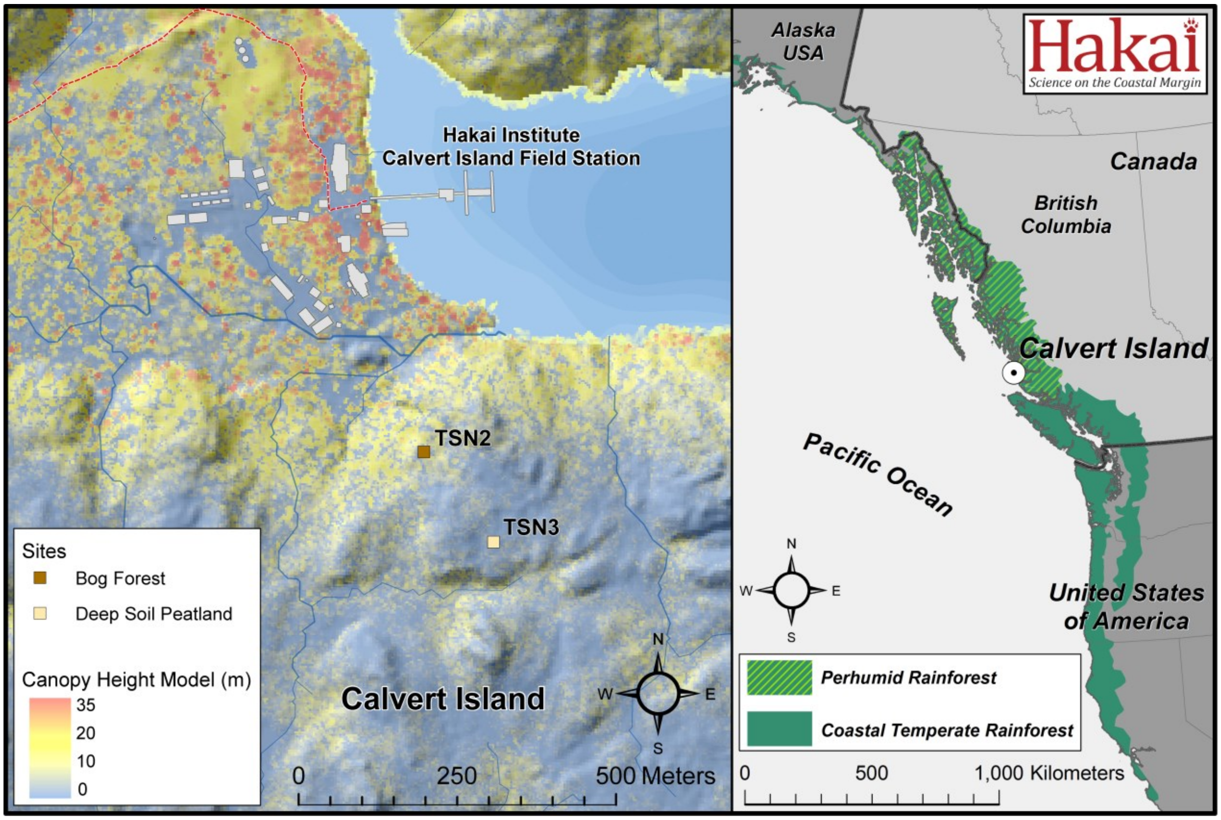

Supplementary Figure 1. Location of the study sites and the Calvert Island Field Station in the Perhumid Pacific Coastal Temperate Rainforest (PCTR). Inset based on data from Wolf et al. (1995) shows location of the Terrestrial Sensor Network (TSN) sites on Calvert Island. The canopy height model imposed on the landscape map indicates tree position and height. The deep soil peatland site (TSN3) is located within an ombrotrophic bog on a plateau above the bog forest site (TSN2), which is itself located on a north-facing slope. These landscape positions are characteristic of bog and forest sites throughout the PCTR. Full interactive map can be found at: data.hakai.org

Supplementary Table 1. DNA and RNA library counts for all samples collected from Bog Forest (TSN2) and Peat Bog (TSN3) sites.

Samples			DNA Libraries			RNA Libraries			
Date	Site	Depth	16S rRNA	ITS	18S rRNA	16S rRNA	ITS	18S rRNA	Meta-transcriptomes
2015-06-16	Bog Forest (TSN2)	10cm	3	3	3	3	3	3	0
2015-06-16	Bog Forest (TSN2)	25cm	3	3	3	3	3	3	0
2015-06-16	Bog Forest (TSN2)	40cm	3	3	3	0	0	0	0
2015-06-16	Peat Bog (TSN3)	10cm	3	3	3	3	3	3	0
2015-06-16	Peat Bog (TSN3)	20cm	3	3	3	3	3	3	0
2015-06-16	Peat Bog (TSN3)	30cm	3	3	3	0	0	0	0
2015-07-23	Bog Forest (TSN2)	10cm	3	3	3	3	3	3	3
2015-07-23	Bog Forest (TSN2)	25cm	3	3	3	3	3	3	0
2015-07-23	Bog Forest (TSN2)	40cm	3	3	3	0	0	0	0
2015-07-23	Peat Bog (TSN3)	10cm	3	3	3	3	3	3	3
2015-07-23	Peat Bog (TSN3)	20cm	3	3	3	3	3	3	0
2015-07-23	Peat Bog (TSN3)	30cm	3	3	3	0	0	0	0
2015-07-25	Bog Forest (TSN2)	10cm	3	3	3	3	3	3	0
2015-07-25	Bog Forest (TSN2)	25cm	3	3	3	3	3	3	0
2015-07-25	Bog Forest (TSN2)	40cm	3	3	3	0	0	0	0
2015-07-25	Peat Bog (TSN3)	10cm	3	3	3	3	3	3	0
2015-07-25	Peat Bog (TSN3)	20cm	3	3	3	2	3	2	0
2015-07-25	Peat Bog (TSN3)	30cm	3	3	3	0	0	0	0
2015-10-28	Bog Forest (TSN2)	10cm	3	3	3	3	3	3	2
2015-10-28	Bog Forest (TSN2)	25cm	3	3	3	3	3	3	0
2015-10-28	Bog Forest (TSN2)	40cm	3	3	3	0	0	0	0
2015-10-28	Peat Bog (TSN3)	10cm	3	3	3	2	2	2	2
2015-10-28	Peat Bog (TSN3)	20cm	3	3	3	3	3	3	0
2015-10-28	Peat Bog (TSN3)	30cm	3	3	3	0	0	0	0
2016-02-26	Bog Forest (TSN2)	10cm	3	2	3	3	3	3	0
2016-02-26	Bog Forest (TSN2)	25cm	3	3	3	3	3	3	0
2016-02-26	Bog Forest (TSN2)	40cm	3	3	3	0	0	0	0
2016-02-26	Peat Bog (TSN3)	10cm	3	3	3	3	3	3	0
2016-02-26	Peat Bog (TSN3)	20cm	3	3	3	3	3	3	0
2016-02-26	Peat Bog (TSN3)	30cm	3	3	3	0	0	0	0
2016-04-16	Bog Forest (TSN2)	10cm	3	3	3	3	3	3	0
2016-04-16	Bog Forest (TSN2)	25cm	3	2	3	3	3	3	0
2016-04-16	Bog Forest (TSN2)	40cm	3	2	3	0	0	0	0
2016-04-16	Peat Bog (TSN3)	10cm	3	3	3	3	3	3	0
2016-04-16	Peat Bog (TSN3)	20cm	3	1	3	3	3	3	0
2016-04-16	Peat Bog (TSN3)	30cm	2	0	2	0	0	0	0

Supplementary Table 2. Results of mixed effects ANOVA for linear models of data and site for gas flux measurements corresponding to soil sampling periods. For CH_4 flux, where there was a significant interaction, ANOVAs to determine date effects were run on flux rate data from individual sites.

CO2 Flux				
	numDF	denDF	F-value	p-value
(Intercept)	1	17	170.152	<.0001
date	4	17	9.01888	0.0004
plot	1	17	0.3651	0.5537
date:site	4	17	0.7823	0.5521
CH4 Flux				
	numDF	denDF	F-value	p-value
(Intercept)	1	17	0.93657	0.3467
date	4	17	0.9517	0.4587
plot	1	17	3.04323	0.0991
date:site	4	17	3.5676	0.0274
Bog Forest (TSN2)				
(Intercept)	1	8	62.5324	<.0001
date	4	8	17.4271	5.00E-04
Peat Bog (TSN3)				
(Intercept)	1	8	1.55609	0.2475
date	4	8	1.91776	0.2009
N2O Flux				
	numDF	denDF	F-value	p-value
(Intercept)	1	16	0.07346	0.7898
date	4	16	0.61571	0.6576
plot	1	16	0.28842	0.5986
date:site	4	16	0.27129	0.8922

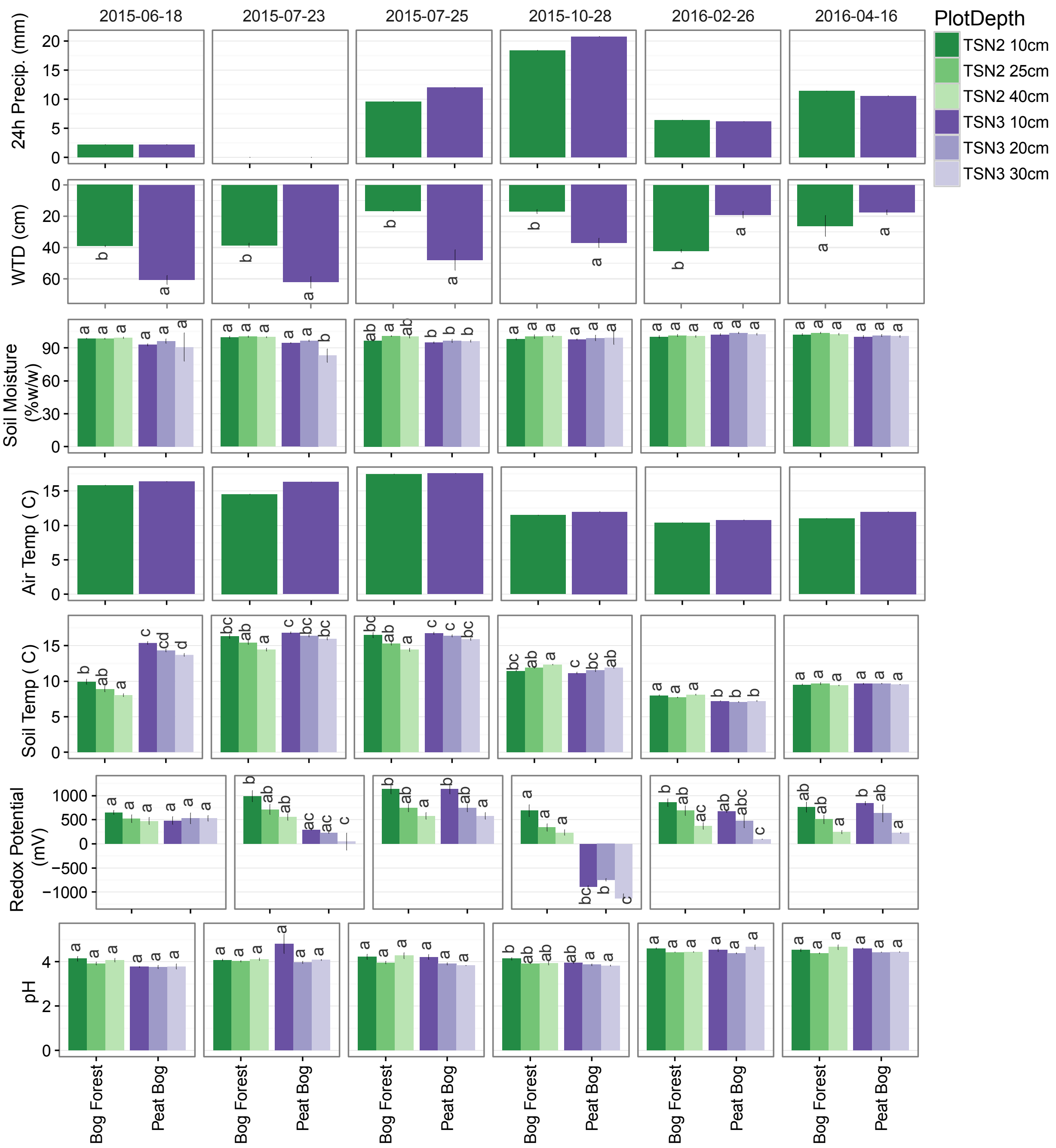

Supplementary Figure 2. Soil and site micro-climactic characteristics in bog forest (TSN2) and peat bog (TSN3) based on sensor readings at time of sampling, with the exception of pH and gravimetric soil moisture. Different letters denote statistical differences at $\alpha = 0.05$. Values and error bars show mean and standard error, respectively (n = 3 for each). Full interactive sensor data can be found at: data.hakai.org

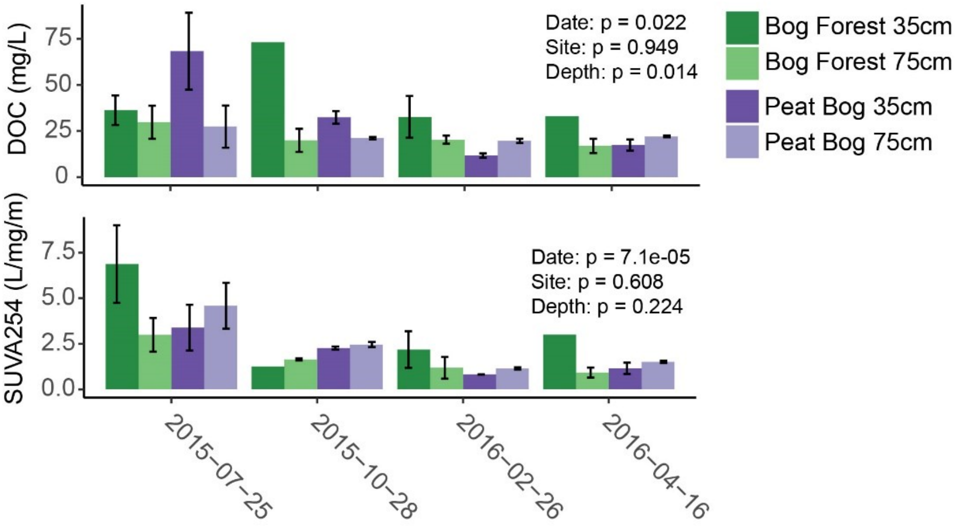

Supplementary Figure 3. Soil water DOC concentration and $SUVA_{254}$. P-values following multi-factor ANOVA provided for each DOC and SUVA. Values and error bars show mean and standard error, respectively (n = 3 for each).

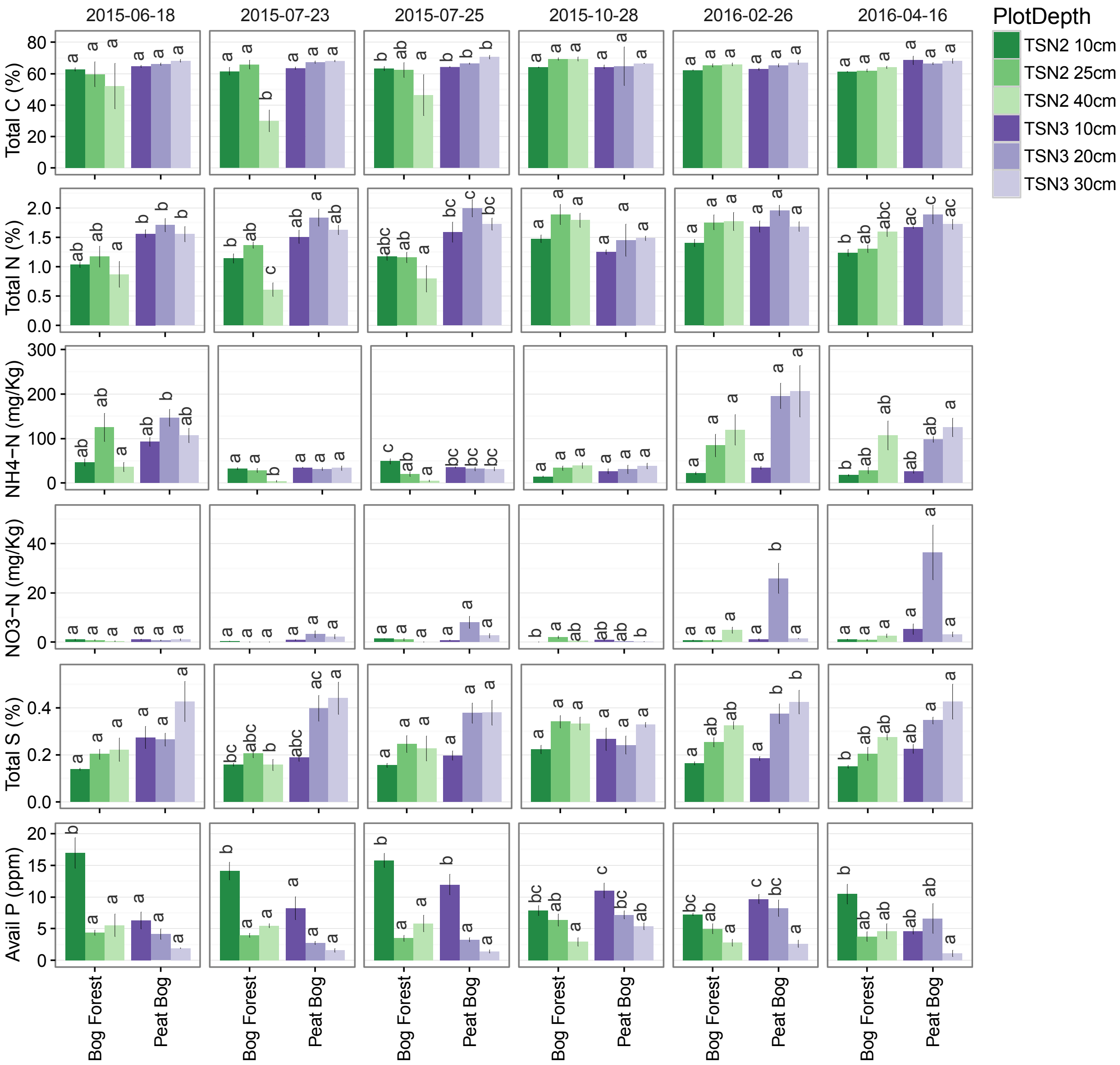

Supplementary Figure 4. Selected soil chemical characteristics in bog forest (TSN2) and peat bog (TSN3). Different letters denote statistical differences at $\alpha = 0.05$. Values and error bars show mean and standard error, respectively (n = 3 for each).

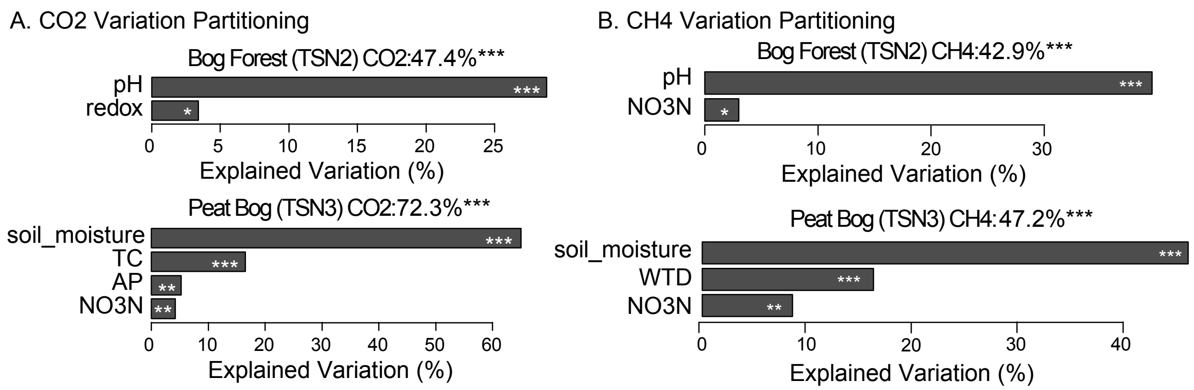

Supplementary Figure 5. Canonical variation partitioning analysis of A) net CO_2 flux rates and B) net CH_4 flux rates in bog forest and peat bog sites. Results of two-way ANOVA provided for each gas. Variation partitioning of gas flux rates from individual sites were constrained by soil micro-meteorological and chemistry variables. Model variables with lowest explanatory power were reduced stepwise until only terms with positive explained variance with $p < 0.05$ remained (*, p<0.05; **, p<0.01; ***, p<0.001).

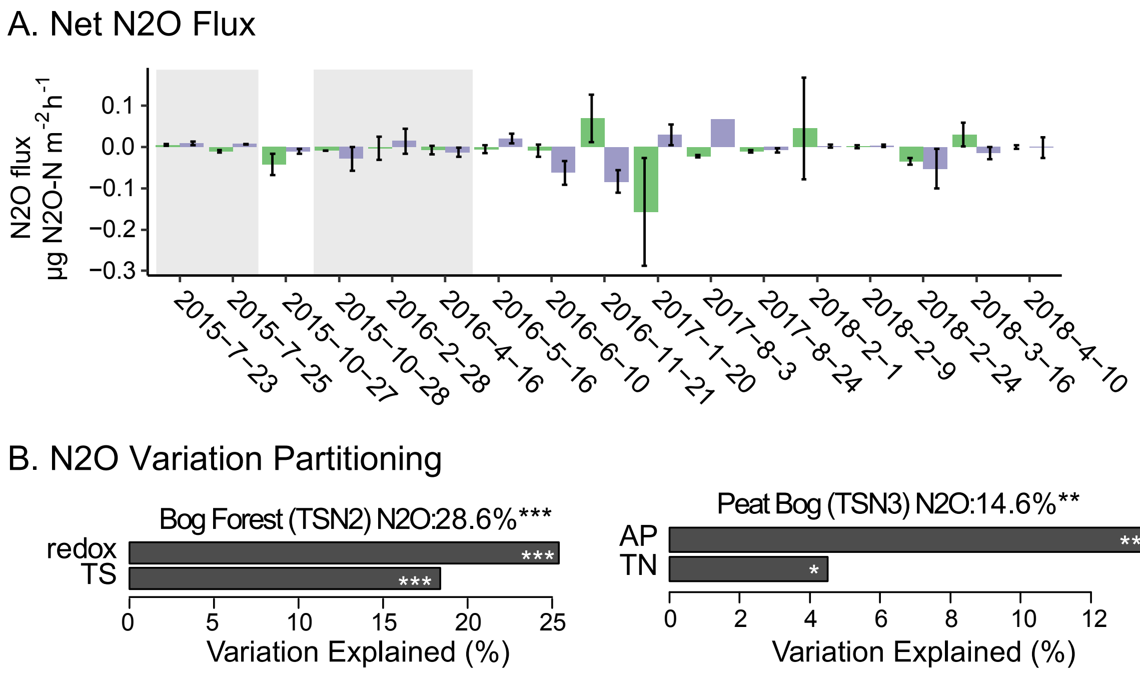

Supplementary Figure 6. Soil net N_2O fluxes in bog forest and peat bog sites and sources of variation. A) Net N_2O flux rates. Values that correspond to microbial sampling dates highlighted in grey. Values and error bars show mean and standard error, respectively (n = 3 for each). B). Canonical variation partitioning analysis as in Supplementary Figure 6.

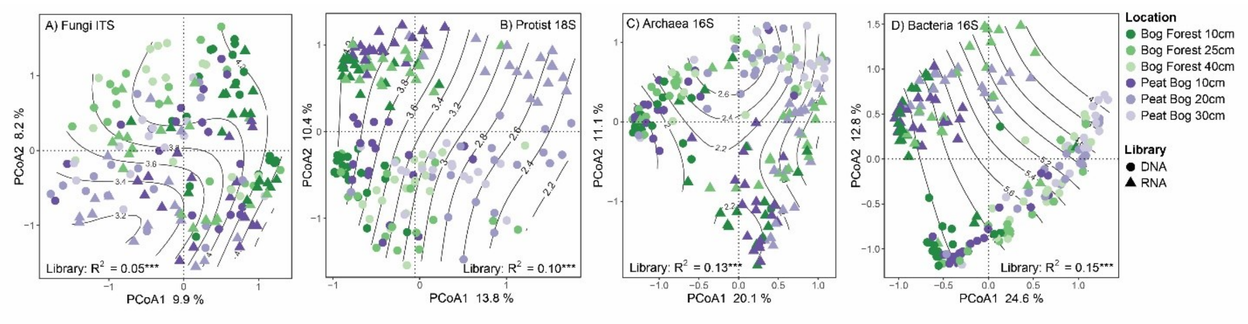

Supplementary Figure 7. Principal coordinate analysis (PCoA) of Bray-Curtis dissimilarly for A) fungal ITS OTUs, b) protist 18S OTUs, c) archaeal 16S OTUs and d) bacterial 16S OTUs from DNA and RNA libraries in Bog Forest (TSN2) and Peat Bog (TSN3) plots. Proportion of variation explained by library type following PERMANOVA with 999 permutations provided (p-value: *, p<0.05; **, p<0.01; ***, p<0.001). Shannon diversity is fit to ordination surfaces as thinplate splines using generalized additive models (GAM).

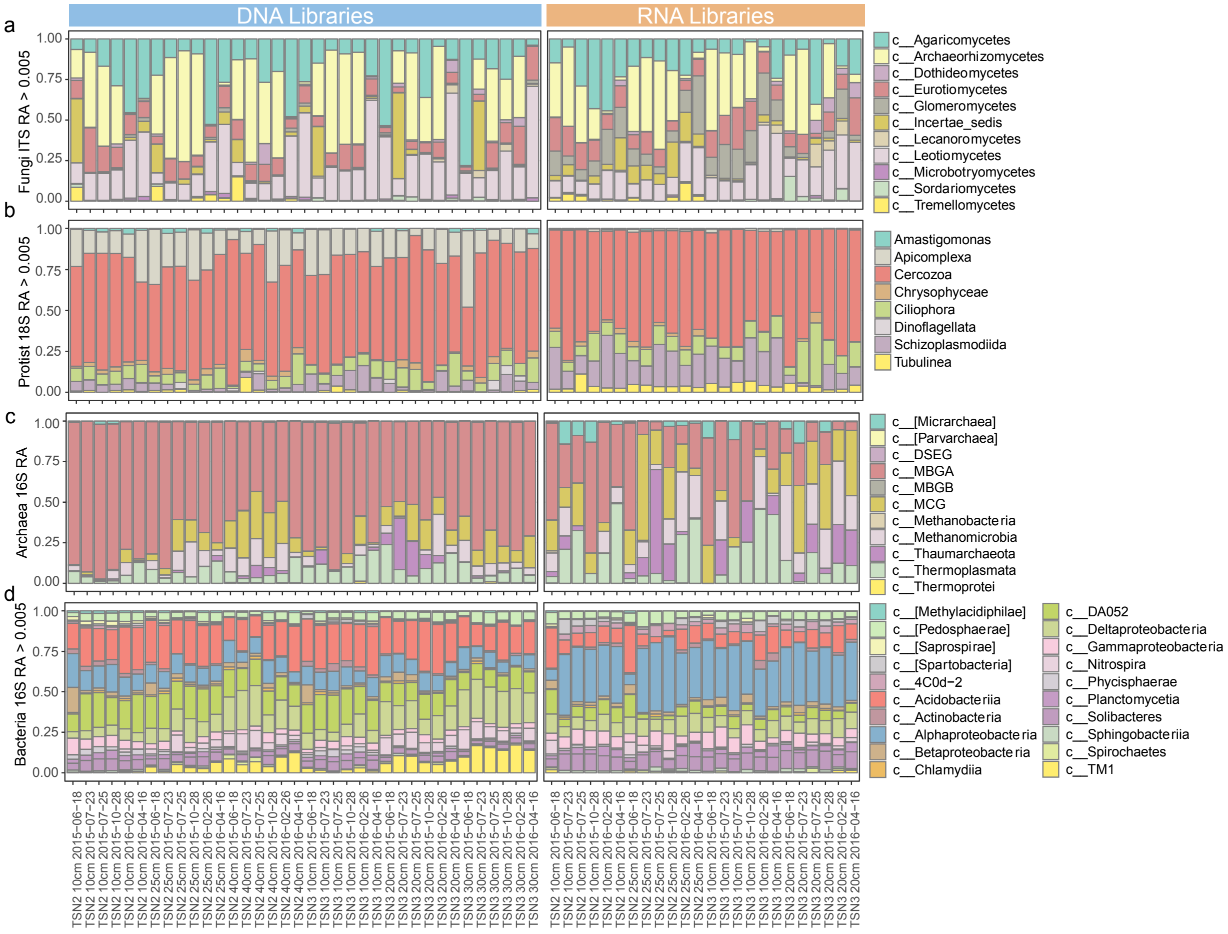

Supplementary Figure 8. Taxonomic identity at the Class level for a) fungal ITS phylotypes and b) 18S rRNA taxonomic identity at “D3” for protist phylotypes, c) archaeal 16S rRNA phylotypes, b) bacterial 16S rRNA phylotypes from DNA and RNA libraries in Bog Forest (TSN2) and Peat Bog (TSN3) plots. Each bar represents the mean of three replicates. Relative abundance (RA) cut-off for bacteria and fungi = 0.005.

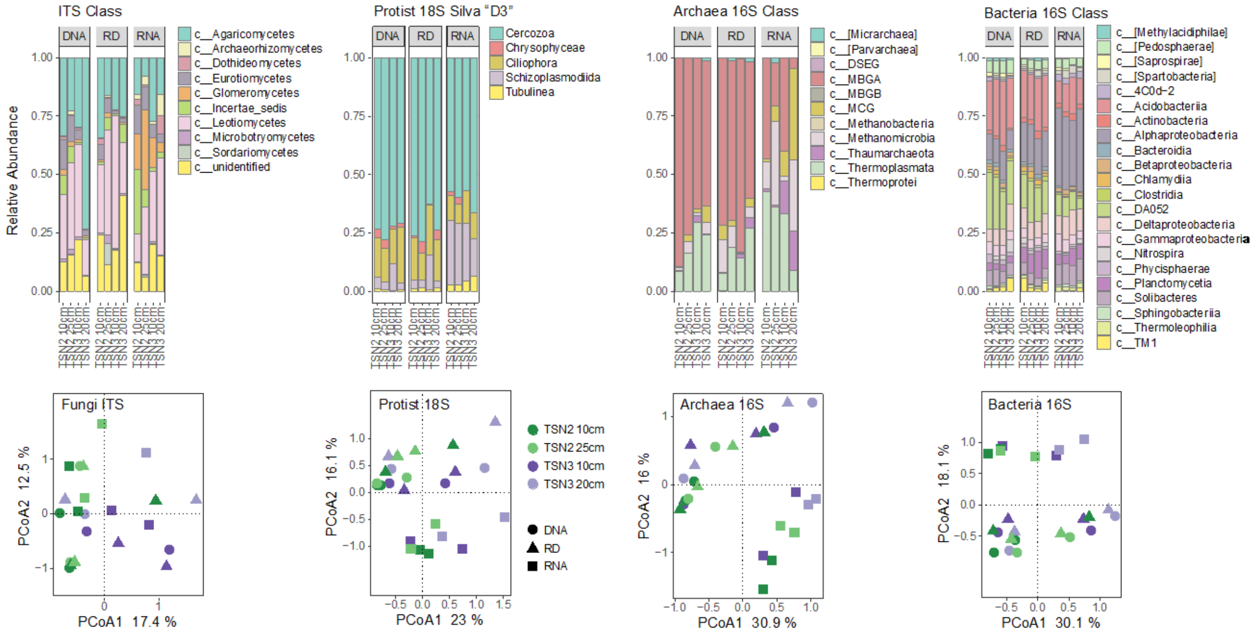

pairs	df	R2	p.adj.
DNA vs. RNA	15	0.14	0.003 *
DNA vs. RD	15	0.08	1
RNA vs. RD	15	0.14	0.003 *

pairs	df	R2	p.adj.
DNA vs. RNA	15	0.19	0.003 *
DNA vs. RD	15	0.08	0.16
RNA vs. RD	15	0.20	0.003 *

pairs	df	R2	p.adj.
DNA vs. RNA	15	0.24	0.006 *
DNA vs. RD	15	0.05	1
RNA vs. RD	15	0.26	0.003 *

pairs	df	R2	p.adj.
DNA vs. RNA	15	0.22	0.003 *
DNA vs. RD	15	0.08	0.78
RNA vs. RD	15	0.20	0.003 *

Supplementary Figure 9. Comparison of extraction methods. DNA and RNA extracted as in methods. RD samples were DNA extracted using RNA extraction chemistry (See Supplementary Methods for information). Top row: taxonomic identity at the Class level protist 18S rRNA, fungal ITS, archaeal 16S rRNA and bacterial 16S rRNA OTUs in Bog Forest (TSN2) and Peat Bog (TSN3) plots. Each bar represents the mean of three replicates. Relative abundance cut-off for bacteria, fungi and protists = 0.005. Middle row: PCoA of Bray-Curtis dissimilarity showing sample location and extraction chemistry. Bottom row: pairwise PERMANOVA comparison of nucleic acid fractions with FDR-adjusted p-values (*, p<0.05; **, p<0.01; ***, p<0.001).

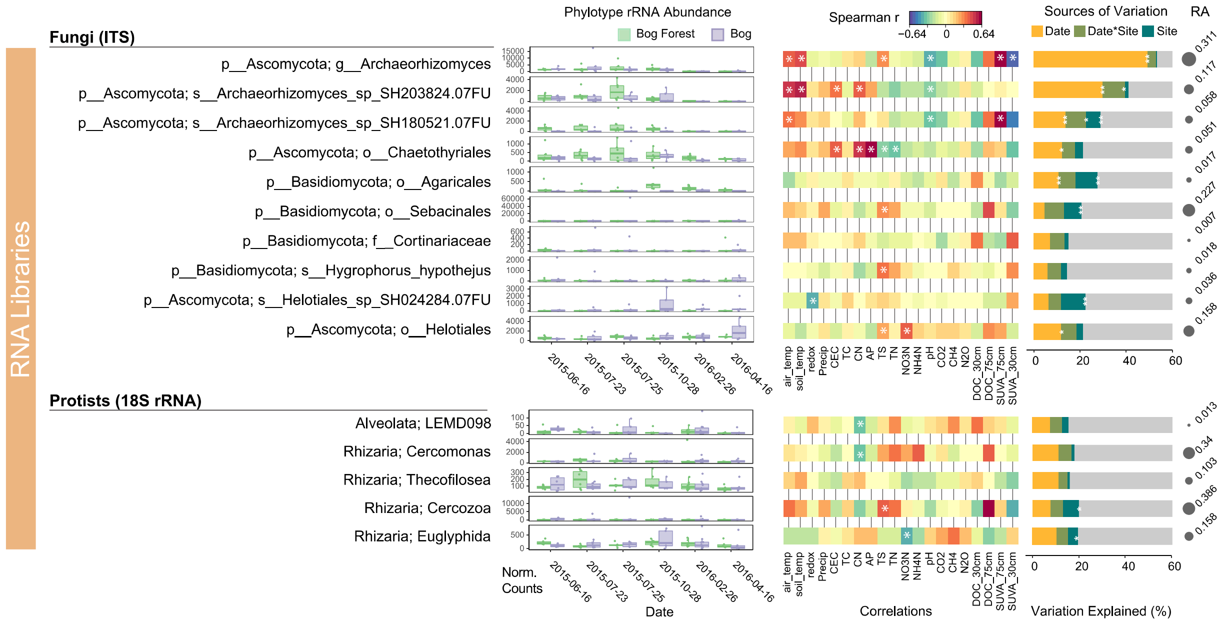

Supplementary Figure 10. Influence of season and ecohydrology on Fungal ITS and Protist 18S rRNA phylotype abundance in RNA libraries. DeSeq2-normalized counts for the 10 most-abundant fungal phylotypes and five most-abundant protist phylotypes in bog forest and peat bog sites shown for each sampling date. Spearman correlations with environmental variables and variation partitioning results provided for each phylotype. FDR-corrected p-values < 0.05 following Spearman correlation are denoted by a single asterisks. Variation partitioning results are denoted by asterisks according to p-value (*, p<0.05; **, p<0.01). Phylotype order determined by complete-linkage clustering of normalized abundance by date. Circle size shows phylotype relative abundance (RA) in RNA libraries.

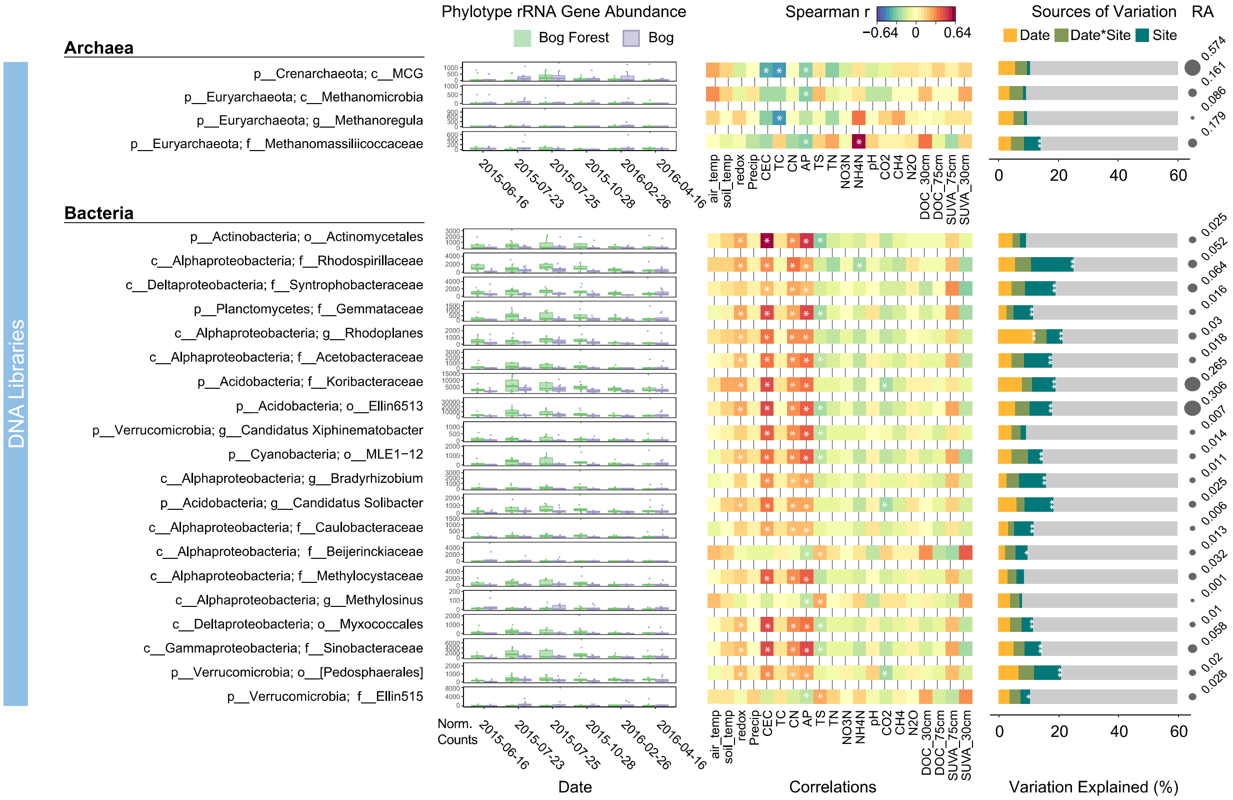

Supplementary Figure 11. Influence of season and ecohydrology on archaeal and bacterial 16S rRNA gene phylotype abundance in DNA libraries. DeSeq2-normalized counts for the four most-abundant archaeal phylotypes and 20 most-abundant bacterial phylotypes in bog forest and peat bog sites shown for each sampling date. Spearman correlations with environmental variables and variation partitioning results provided for each phylotype FDR-corrected p-values < 0.05 following Spearman correlation are denoted by a single asterisks. Variation partitioning results are denoted by asterisks according to p-value (*, p<0.05; **, p<0.01). Phylotype order determined by complete-linkage clustering of normalized abundance by date. Circle size shows phylotype relative abundance (RA) in DNA libraries.

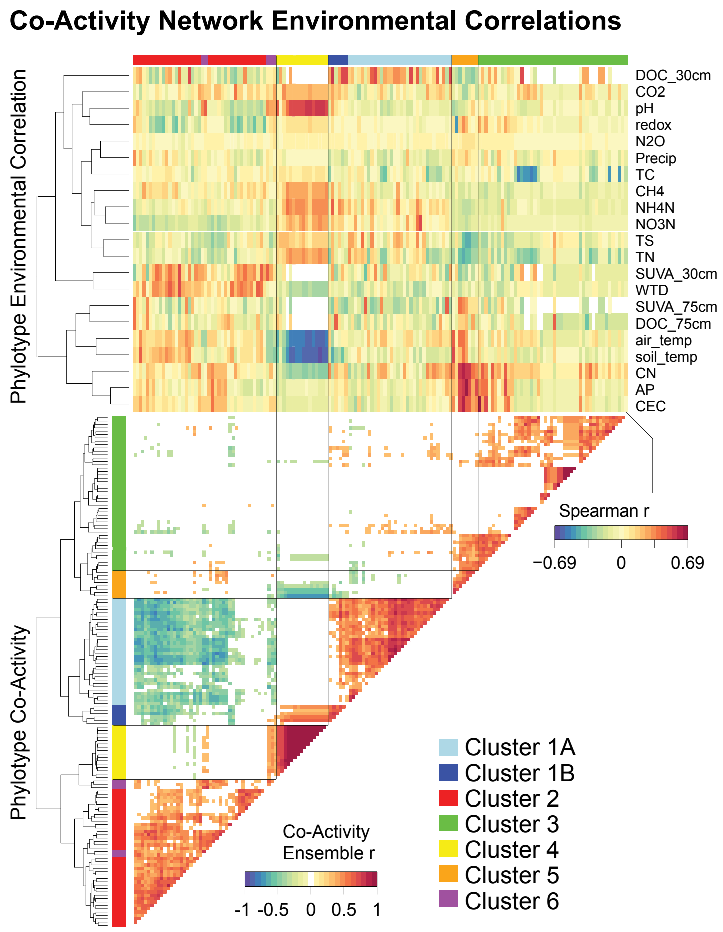

Supplementary Figure 12. Environmental correlations of phylotype co-activity clusters (Figure 5). Ensemble correlations used to build network models in Figure 5 are shown in the lower panel. Clusters are determined by complete linkage clustering using *hclust* and are coded by color. Only taxa correlations with $p > 0.001$ are shown.

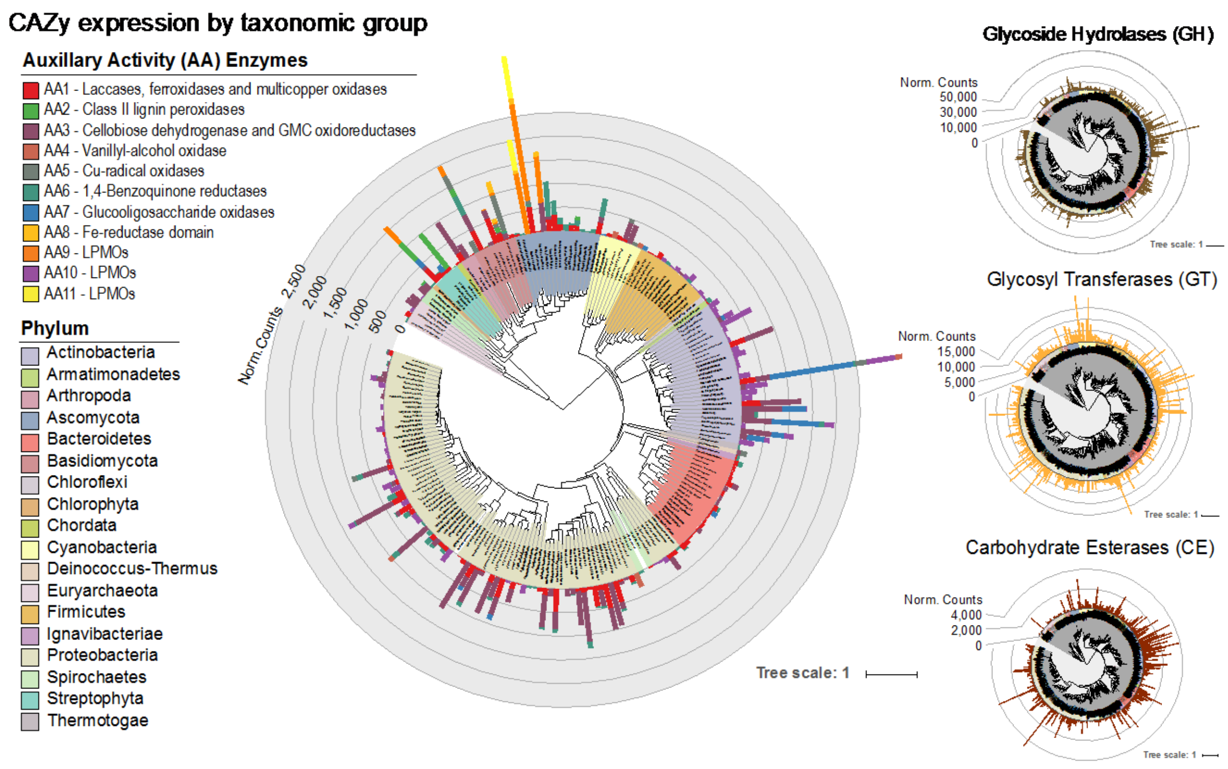

Supplementary Figure 13. Phylogenetic distribution of transcripts from CAZy classes in metatranscriptomes from peat bog and bog forest soils (depth: 10cm). Maximum-likelihood tree build using MUSCLE alignments of ribosomal SSU sequences with FastTree based on taxonomic annotation. Colors extending from tree nodes indicate phylum membership. Barplot colors extending beyond trees indicate abundance as DeSeq2-normalized counts mapped to 16S and 18S rRNA phylogenetic trees using iTOL v3. AA, Auxiliary Activity; CE, Carbohydrate Esterase; GH, Glycoside Hydrolase; GT, Glycosyl Transferase. Full, interactive AA tree (https://itol.embl.de/tree/12818967504543150004 6186); GH, GT, CE tree (https://itol.embl.de/tree/1281897043473551498083538).
