# Supplementary material for: Seasonal and ecohydrological regulation of active microbial populations involved in DOC, CO2, and CH4 fluxes in temperate rainforest soil
Source: ISME J. 2018 Dec 11;13(4):950–63. doi: 10.1038/s41396-018-0334-3 (PMC6461783; doi:10.1038/s41396-018-0334-3)
Supplement: Supplementary file 2 — Supplemental Methods [file 41396_2018_334_MOESM2_ESM.pdf]

Supplementary Methods: Seasonal and ecohydrological regulation of active microbial populations involved in DOC, CO_2 and CH_4 fluxes in temperate rainforest soil

David J. Levy-Booth[1,2], Ian J.W. Giesbrecht[2,3], Colleen T.E. Kellogg[1,2], Thierry J. Heger[,4], David V. D'Amore[5], Patrick J. Keeling[6], Steven J. Hallam[1], William W. Mohn[1]*

Affiliations:

1. Department of Microbiology & Immunology, Life Sciences Institute, University of British Columbia, Vancouver, British Columbia, Canada
2. Hakai Institute, Tula Foundation, Heriot Bay, British Columbia, Canada
3. School of Resource and Environmental Management, Simon Fraser University, Burnaby, British Columbia, Canada
4. The University of Applied Sciences Western Switzerland, CHANGINS, Switzerland
5. U.S. Department of Agriculture, Forest Service, Pacific Northwest Research Station, Juneau, Alaska, USA
6. Department of Botany, University of British Columbia, Vancouver, British Columbia, Canada

*Correspondence: WW Mohn, Department of Microbiology & Immunology, Life Sciences Institute, University of British Columbia, 2350 Health Sciences Mall, Vancouver, British Columbia V6T 1Z3, Canada. E-mail: wmohn@mail.ubc.ca

Soil water

Soil water was sampled from 30cm diameter pan lysimeters with 200 micron polypropylene mesh (at 30 cm) and 5cm diameter piezometers (at 75 cm), and syringe-filtered with aqueous 20 µm polycarbonate (Whatman plc, Maidstone, UK) in each subplot. Aliquots were partitioned for DOC concentration and specific ultraviolet absorbance (SUVA) at 254nm of DOC using an Aqualog spectrofluorometer (Horiba Canada Inc., London, CAN).

Sample collection and library preparation

Two-hundred and sixteen soil samples were collected for this study. Of these, 108 DNA libraries, 72 RNA libraries were prepared for 16S rRNA, ITS and 16S rRNA amplicon sequencing (Supplemental Table 1). In addition, successful metatranscriptomes were recovered from three bog forest and three peat bog samples from July 23, 2015, as well as two bog forest and two peat bog metatranscriptomes from October 28, 2015.

To test if extraction method influenced microbial community structure, the DNA fraction of eight soil samples from April 18, 2016 from both bog and forest sites were extracted for total nucleic acids using the RNA extraction protocol were purified using the PowerClean Pro DNA Clean-Up Kit (MoBio Laboratories, Inc., Carlsbad, U.S.A.). The RNA-protocol-DNA-fraction (RD) samples were sequenced as described in the main text. Statistical comparisons between nucleic acid fractions were made using the *pairwise.adonis* extension (https://github.com/pmartinezarbizu/pairwiseAdonis) of the *adonis* function in the *vegan* package for R. Results are shown in Supplementary Figure

Sequencing

Library preparation used SequalPrep normalization (Thermo Fisher Scientific Inc.) 250-bp paired-end (PE) sequencing was performed on the MiSeq platform (Illumina, Inc. San Diego, U.S.A.) using a 300-cycle V2 MiSeq Reagent Kit.

Bioinformatics

Paired-end joining of raw Illumina fastq iTag reads used FLASh 1.2.11 (Magoč and Salzberg, 2011) and quality filtering used USEARCH 9.0.2132 (Edgar and Flyvbjerg, 2015). Reads truncated before the first base with a quality score below Q20 and were only retained if the length of the filtered read was at least 75% of input sequence length. No ambiguous base calls were permitted. Paired-end joining and quality filtering each removed ~10% of sequences from each sample. Chimera detection used UCHIME 4.2.40 with default parameters (Edgar et al., 2011). OTU selection and binning used the open reference pipeline in QIIME 1.9.1 (Caporaso et al., 2010b) using the UCLUST algorithm (Edgar, 2010). 16S OTUs were binned using Greengenes 13_5 with an identity threshold of 97%, followed by *de novo* clustering of unidentified sequences. 18S OTUs were selected at 97% identity against the Silva 108 Eukaryotes database. Sequences were filtered and aligned with PyNast (Caporaso et al., 2010a). Singletons were removed for downstream analysis. Fungal ITS sequence processing used the default PIPITS pipeline (Gweon et al., 2015). PIPITS uses PEAR 0.9.6 (Zhang et al., 2014) for paired-end joining and the fastx-toolkit (Hannon, http://hannonlab.cshl.edu) for quality filtering. ITSX 1.0.11 (Bengtsson-Palme et al., 2013) was used to extract ITS sequences and VSEARCH 1.10.2 (Rognes,

https://github.com/torognes/vsearch/) was used to remove chimeras and bin OTUs against the UNITE 7.0 database (Kõljalg et al., 2013) with RDP classifier 2.10 (Wang et al., 2007).

Bacterial and archaeal 16S rRNA (RNA library) and rRNA gene (DNA library) amplicons targeting total and active communities, respectively, yielded 10,082,650 reads from 108 soil samples after paired-end joining. Following filtering, 7,673,915 reads clustered as 116,555 OTUs with a mean (standard deviation) of 33,220 (21,381) OTUs per sample. Of the 16S rRNA OTUs, 777 were archaeal, representing 2.1% of 16S rRNA counts. Fungi ITS amplicon sequencing resulted in 13,617,223 total sequences, 11,777,089 filtered sequences and 11,915 distinct OTUs. Mean OTU counts per sample were 39,937 (31,420). Eukaryotic 18S rRNA amplicon sequencing resulted in 14,575,071 total and 10,243,426 filtered sequences and 28,375 OTUs with 33,695 (22,840) OTUs per sample.

Analysis of seasonal and site related trends in microbial populations used phylotypes, which indicates the lowest taxonomic classification for a set of reads (often incorporating multiple OTUs). At this level, 10 most abundant fungal phylotypes, five most-abundant protist phylotypes, four most abundant archaeal phylotypes and 20 most abundant bacterial phylotypes were selected for further analysis. Phylotype networks were built using Fruchterman-Reingold layouts using *igraph* and plotted in *Cytoscape* 2.8 (Smoot et al., 2011).

Metatranscriptome reads were quality checked and trimmed using Trimmomatic 0.3.6 (Bolger et al., 2014) with a minimum length of 70 bp and a minimum 4-base sliding quality window of Q20. Further rRNA and PhiX removal used sortmerna 2.1 (Kopylova et al., 2012). Paired reads were joined with Fastq-Join (http://code.google.com/p/ea-utils) using a minimum overlap of 8 bp and a maximum difference of 10%. FragGeneScan 1.17 (Rho et al., 2010) was used to predict putative gene sequences, which were clustered as amino acid sequences using CD-HIT v4.6 (Fu et al., 2012) with an identity threshold of >= 95% and a gene overlap of >= 90, giving an median read length of 56 ± 25 amino acids. Amino acid sequences were annotated using DIAMOND 0.7.1 (Buchfink et al., 2015) against the NCBI nr database (2015-25-05) and KEGG Orthology (KO) database (Feb2015X) with an *E*-value threshold of 1E−5 (Cardenas et al., 2015). Carbohydrate-Active enZYmes (CAZy) sequences were selected using Lombard et al. (2014). MEGAN 6 (Huson et al., 2016) was used for lowest common ancestor (LCA) assignment and expanded to higher ranks using *taxize* in R 3.2.4.

In parallel, trimmed and filtered paired-end reads were assembled using Trinity 2.4.0 (Grabherr et al., 2011) prior to annotation. Trinity assembly resulted in 2,066,189 assembled transcripts with a mean (standard deviation) length of 310bp (194bp). However, only 2.8% of read pairs were assembled and few assembled transcripts could be mapped to reference sequences using BowTie2. Therefore, subsequent analysis was performed using short read annotation as in Cardenas et al. (2015).

Shotgun metatranscriptome sequencing resulted in a mean of 59,516,349 raw 125bp-PE reads for 10 samples that passed QC. Samples > 10 cm soil depth did not yield adequate mRNA and could not be adequately purified of organic co-extractants. 14.4% of reads were removed during QC, trimming, in silico rRNA sequence removal and paired-end joining. Of these, 1,211 unique taxonomic identities were assigned to 5,156,732 reads and 11,202 unique KEGG orthology (KO) identifiers were assigned to 6,113,235 reads.

Statistical analysis

Partial regression of variables grouped into soil chemistry, soil climate, DOC and gas fluxes to partition variation in spatial (ecohydrological site, soil depth) and temporal (date). Based on this initial analysis, variables were grouped either spatially or temporally for presentation in Figure 1B. Differences between factors in contrasting sampling dates representing summer (2015-07-23) and winter (2016-02-28) were assed using simple t-tests. One-way ANOVA with Tukey post-hoc testing was used to determine if these variable groupings differed at $\alpha < 0.05$. Full data with one-way ANOVA with Tukey post-hoc testing at each date are presented as supplementary figures. Gas flux data were analyzed with a two-way ANOVA by site and date. Further, partial regression was used to select variables uniquely explaining the largest portion variation. Full models containing all microbial, climactic and chemical variables were reduced stepwise by removing variables that did not contribute positively to the model. Soil water DOC concentrations and $SUVA_{254}$ values were analyzed with a multi-factor ANOVA by site, date and depth. Microbial phylotypes following amplicon sequencing from DNA and RNA libraries were ordinated using Bray-Curtis distance-based redundancy analysis (db-RDA) against soil chemical and climate variables. Ordinations, PERMANOVA, canonical variation partitioning and calculation of Shannon diversity indices used *vegan* 2.4.3 (Oksanen et al., 2017). Phylotype environmental Spearman correlation *p*-values were calculated using the *rcorr* function in the *Hmisc* package, and false-discovery-rate (FDR) corrected using the *p.adjust* function in R. All plots were constructed using *ggplot2* 2.2.1 unless noted. Phylogenetic trees were based on ribosomal protein trees from Hug et al. (2016), filtered by CAZy-containing, or KEGG-marker containing organisms and constructed using iTOL v3 (Letunic and Bork, 2016).

References

Bengtsson-Palme, J., Ryberg, M., Hartmann, M., Branco, S., Wang, Z., Godhe, A., De Wit, P., Sánchez-García, M., Ebersberger, I., de Sousa, F., Amend, A., Jumpponen, A., Unterseher, M., Kristiansson, E., Abarenkov, K., Bertrand, Y.J.K., Sanli, K., Eriksson, K.M., Vik, U., Veldre, V., Nilsson, R.H., 2013. Improved software detection and extraction of ITS1 and ITS2 from ribosomal ITS sequences of fungi and other eukaryotes for analysis of environmental sequencing data. Methods Ecol. Evol. 4, 914–919. doi:10.1111/2041-210X.12073

Bolger, A.M., Lohse, M., Usadel, B., 2014. Trimmomatic: a flexible trimmer for Illumina sequence data. Bioinforma. Oxf. Engl. 30, 2114–2120. doi:10.1093/bioinformatics/btu170

Buchfink, B., Xie, C., Huson, D.H., 2015. Fast and sensitive protein alignment using DIAMOND. Nat. Methods 12, 59–60. doi:10.1038/nmeth.3176

Caporaso, J.G., Bittinger, K., Bushman, F.D., DeSantis, T.Z., Andersen, G.L., Knight, R., 2010a. PyNAST: a flexible tool for aligning sequences to a template alignment. Bioinforma. Oxf. Engl. 26, 266–267. doi:10.1093/bioinformatics/btp636

Caporaso, J.G., Kuczynski, J., Stombaugh, J., Bittinger, K., Bushman, F.D., Costello, E.K., Fierer, N., Peña, A.G., Goodrich, J.K., Gordon, J.I., Huttley, G.A., Kelley, S.T., Knights, D., Koenig, J.E., Ley, R.E., Lozupone, C.A., McDonald, D., Muegge, B.D., Pirrung, M., Reeder, J., Sevinsky, J.R., Turnbaugh, P.J., Walters, W.A., Widmann, J., Yatsunenko, T., Zaneveld, J., Knight, R., 2010b. QIIME allows analysis of high-throughput community sequencing data. Nat. Methods 7, 335–336. doi:10.1038/nmeth.f.303

Cardenas, E., Kranabetter, J.M., Hope, G., Maas, K.R., Hallam, S., Mohn, W.W., 2015. Forest harvesting reduces the soil metagenomic potential for biomass decomposition. ISME J. 9, 2465–2476. doi:10.1038/ismej.2015.57

Edgar, R.C., 2010. Search and clustering orders of magnitude faster than BLAST. Bioinforma. Oxf. Engl. 26, 2460–2461. doi:10.1093/bioinformatics/btq461

Edgar, R.C., Flyvbjerg, H., 2015. Error filtering, pair assembly and error correction for next-generation sequencing reads. Bioinforma. Oxf. Engl. 31, 3476–3482. doi:10.1093/bioinformatics/btv401

Edgar, R.C., Haas, B.J., Clemente, J.C., Quince, C., Knight, R., 2011. UCHIME improves sensitivity and speed of chimera detection. Bioinforma. Oxf. Engl. 27, 2194–2200. doi:10.1093/bioinformatics/btr381

Fu, L., Niu, B., Zhu, Z., Wu, S., Li, W., 2012. CD-HIT: accelerated for clustering the next-generation sequencing data. Bioinforma. Oxf. Engl. 28, 3150–3152. doi:10.1093/bioinformatics/bts565

Grabherr, M.G., Haas, B.J., Yassour, M., Levin, J.Z., Thompson, D.A., Amit, I., Adiconis, X., Fan, L., Raychowdhury, R., Zeng, Q., Chen, Z., Mauceli, E., Hacohen, N., Gnirke, A., Rhind, N., di Palma, F., Birren, B.W., Nusbaum, C., Lindblad-Toh, K., Friedman, N., Regev, A., 2011. Full-length transcriptome assembly from RNA-Seq data without a reference genome. Nat. Biotechnol. 29, 644–652. doi:10.1038/nbt.1883

Gweon, H.S., Oliver, A., Taylor, J., Booth, T., Gibbs, M., Read, D.S., Griffiths, R.I., Schonrogge, K., 2015. PIPITS: an automated pipeline for analyses of fungal internal transcribed spacer sequences from the Illumina sequencing platform. Methods Ecol. Evol. 6, 973–980. doi:10.1111/2041-210X.12399

Hug, L.A., Baker, B.J., Anantharaman, K., Brown, C.T., Probst, A.J., Castelle, C.J., Butterfield, C.N., Hernsdorf, A.W., Amano, Y., Ise, K., Suzuki, Y., Dudek, N., Relman, D.A., Finstad, K.M., Amundson, R., Thomas, B.C., Banfield, J.F., 2016. A new view of the tree of life. Nat. Microbiol. 1, nmicrobiol201648. doi:10.1038/nmicrobiol.2016.48

Huson, D.H., Beier, S., Flade, I., Górska, A., El-Hadidi, M., Mitra, S., Ruscheweyh, H.-J., Tappu, R., 2016. MEGAN Community Edition - Interactive Exploration and Analysis of Large-Scale Microbiome Sequencing Data. PLOS Comput. Biol. 12, e1004957. doi:10.1371/journal.pcbi.1004957

Kõljalg, U., Nilsson, R.H., Abarenkov, K., Tedersoo, L., Taylor, A.F.S., Bahram, M., Bates, S.T., Bruns, T.D., Bengtsson-Palme, J., Callaghan, T.M., Douglas, B., Drenkhan, T., Eberhardt, U., Dueñas, M., Grebenc, T., Griffith, G.W., Hartmann, M., Kirk, P.M., Kohout, P., Larsson, E., Lindahl, B.D., Lücking, R., Martín, M.P., Matheny, P.B., Nguyen, N.H., Niskanen, T., Oja, J., Peay, K.G., Peintner, U., Peterson, M., Põldmaa, K., Saag, L., Saar, I., Schüßler, A., Scott, J.A., Senés, C., Smith, M.E., Suija, A., Taylor, D.L., Telleria, M.T., Weiss, M., Larsson, K.-H., 2013. Towards a unified paradigm for sequence-based identification of fungi. Mol. Ecol. 22, 5271–5277. doi:10.1111/mec.12481

Kopylova, E., Noé, L., Touzet, H., 2012. SortMeRNA: fast and accurate filtering of ribosomal RNAs in metatranscriptomic data. Bioinforma. Oxf. Engl. 28, 3211–3217. doi:10.1093/bioinformatics/bts611

Letunic, I., Bork, P., 2016. Interactive tree of life (iTOL) v3: an online tool for the display and annotation of phylogenetic and other trees. Nucleic Acids Res. 44, W242-245. doi:10.1093/nar/gkw290

Lombard, V., Golaconda Ramulu, H., Drula, E., Coutinho, P.M., Henrissat, B., 2014. The carbohydrate-active enzymes database (CAZy) in 2013. Nucleic Acids Res. 42, D490-495. doi:10.1093/nar/gkt1178

Magoč, T., Salzberg, S.L., 2011. FLASH: fast length adjustment of short reads to improve genome assemblies. Bioinforma. Oxf. Engl. 27, 2957–2963. doi:10.1093/bioinformatics/btr507

Oksanen, J., Blanchet, G., Friendly, M., Kindt, R., Legendre, P., McGlinn, D., 2017. vegan: Community Ecology Package. R package version 2.4-3.

Rho, M., Tang, H., Ye, Y., 2010. FragGeneScan: predicting genes in short and error-prone reads. Nucleic Acids Res. 38, e191. doi:10.1093/nar/gkq747

Wang, Q., Garrity, G.M., Tiedje, J.M., Cole, J.R., 2007. Naïve Bayesian Classifier for Rapid Assignment of rRNA Sequences into the New Bacterial Taxonomy. Appl. Environ. Microbiol. 73, 5261–5267. doi:10.1128/AEM.00062-07

Zhang, J., Kobert, K., Flouri, T., Stamatakis, A., 2014. PEAR: a fast and accurate Illumina Paired-End reAd mergeR. Bioinforma. Oxf. Engl. 30, 614–620. doi:10.1093/bioinformatics/btt593
